# VIS–NIR–SWIR Proximal Spectroradiometry Coupled with Machine Learning and Deep Learning for Ornamental Plant Identification and Classification

**DOI:** 10.1021/acsomega.6c05120

**Published:** 2026-09-14

**Authors:** Renan Falcioni, José Alexandre M. Demattê, Marcos Rafael Nanni

**Affiliations:** † Graduate Program in Agronomy, State University of Maringá, Av. Colombo, 5790, Maringá 87020−900, Paraná, Brazil; ‡ Department of Soil Science, Luiz de Queiroz College of Agriculture, University of São Paulo, Av. Pádua Dias, 11, Piracicaba 13418−900, São Paulo, Brazil

## Abstract

Non-destructive classification of ornamental plant material could improve greenhouse quality control, cultivar screening, and spectral phenotyping; however, most routine decisions still rely on visual inspection. We evaluated proximal VIS–NIR–SWIR spectroradiometry (400–2400 nm) for 900 balanced plant-level leaf or bract spectra representing nine ornamental classes from pothos, poinsettia, geranium, and hibiscus. The spectra formed a highly structured low-dimensional dataset, with the first three principal components explaining 97.11% of the total variance. Full-spectrum and edge-trimmed representations preserved high performance (best macro-F1 = 82.56% and 81.99%, respectively), whereas a ReliefF-selected 16-band green window (547–562 nm) reduced performance to 60.77% macro-F1. Among the full-spectrum deep models, MLP_Deep achieved 80.99% F1. These results show that proximal reflectance enables effective and interpretable plant-level ornamental phenotype classification and discrimination within the present benchmark, whereas compact green-band selection alone cannot replace broader VIS–NIR–SWIR information for closely related foliage classes.

## Graphical abstract

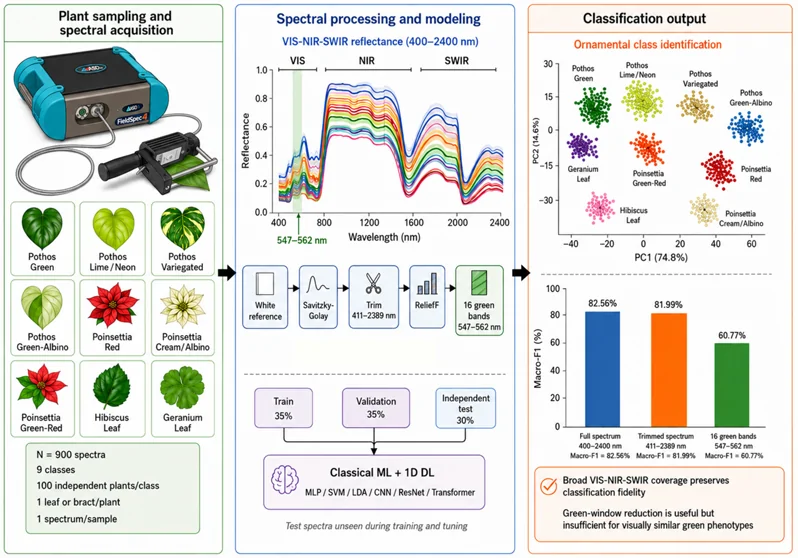

## 1 Introduction

Ornamental horticulture increasingly requires rapid, non-destructive and scalable ways to distinguish taxa, color phenotypes and market states because nursery production, postharvest sorting and cultivar management still depend heavily on visual appraisal. This limitation is especially acute when classes differ mainly in pigment distribution, disease in sectorial variegation or transitional bract color rather than in gross morphology. Proximal spectroscopy offers an attractive alternative because it is fast, portable and compatible with high-throughput phenotyping workflows already being developed for horticulture and floriculture.^1–6^

At the leaf scale, reflectance is the optical outcome of pigment absorption, internal scattering, water absorption and tissue structure.^7–9^ Visible wavelengths are strongly shaped by chlorophylls, carotenoids and anthocyanins; the red edge captures the transition between absorption and scattering, and the short-wavelength infrared band is sensitive to water, dry matter and internal structure.^10–12^ These relationships are well established in the field of leaf optics theory and make proximal VIS–NIR–SWIR measurements especially suitable for ornamental phenotypes defined by green, lime, albino, variegated and red tissues.^7–12^

Recent plant spectroscopy studies have shown that leaf reflectance can capture biochemical, physiological, diseases, taxonomic and genotype-linked variation across controlled and field settings when target traits are explicitly calibrated or when spectral signatures are used for discrimination.^13–15^ Spectral libraries and multispecies analyses likewise show that leaf signatures remain informative across broad functional groups when calibration and transferability are handled carefully. This literature suggests that ornamental material should be amenable to reflectance-based discrimination, although relatively few studies have framed ornamental classification as an interpretable multiclass problem spanning both leaf and bract phenotypes.^14–18^

Interpretability matters because operational deployment is rarely limited by raw accuracy alone. In horticultural settings, users need to know which wavelength regions support discrimination, whether those regions align with plausible pigment or water-sensitive mechanisms, and how much performance is lost when the spectral design is reduced for cheaper hardware.^13–18^ Best-practice work therefore emphasizes coordinated preprocessing, spectral quality control and biologically meaningful band selection rather than purely black-box classification.^19–21^

Machine learning is the natural interface between high-dimensional spectra and actionable decisions, but model ranking often reveals as much of the signal as the headline score does.^4,5,19–21^ Linear margins, distance-based methods, tree-based models and feature-ranking procedures remain highly competitive in spectroscopy when discriminative information is smooth and redundant across adjacent wavelengths.^22–25^ Although deep learning can add value, its benefit depends strongly on the data scale and representation and whether the chosen architecture matches the statistical structure of the spectrum.^26,27^

An additional unresolved issue is how far compact band sets can replace full spectral coverage in realistic classification tasks.^28–32^ Reduced-band designs are desirable for hardware translation, but aggressive compression can discard complementary information in many plant-structures, with leaves, bract, seeds, stem, root distributed across the visible, NIR and SWIR domains.^28–32^ In ornamental plant-structures, where related green foliage phenotypes coexist with pigment-rich bract states, this trade-off has direct consequences for sensor design and nursery-level reliability.^3,6,28–32^

In this study, we analyzed 900 leaf-plant-level samples spanning nine ornamental classes drawn from pothos, poinsettia, geranium and hibiscus plants and evaluated full-spectrum, edge-trimmed and 16-band reduced representations through an interpretable workflow combining class-mean spectra, PCA, one-versus-rest association mapping, hierarchical similarity analysis, ReliefF ranking, classical machine learning and one-dimensional deep learning. Our central hypothesis was that ornamental classes defined by species, organ identity and visible phenotype would carry reproducible VIS–NIR–SWIR reflectance signatures that can be recognized from independent plant-level spectra but that a narrow green-domain band set would not retain enough complementary visible, red-edge, NIR and SWIR information to resolve spectrally overlapping green phenotypes. We therefore asked three main questions: (i) whether ornamental classes can be distinguished from 400–2400 nm reflectance; (ii) whether the most informative wavelengths are concentrated in a biologically plausible subset of the spectrum; and (iii) how much classification fidelity is retained when the representation is compressed to a compact green-domain band set.

## 2 Materials and Methods

### 2.1 Plant Material, Phenotypic Classes and Experimental Design

Nine ornamental classes were analyzed: Albino, Geranium, Green, Green-Albino, Green–Red, Hibisco, Lime, Red and Variegated. The benchmark intentionally combined species-level leaf classes with within-species color phenotypes. Four classes were derived from *Epipremnum aureum* foliage: green (uniform dark-green, Jade-type leaves), lime (uniform lime-green, Neon-type leaves), variegated (green–yellow-cream marbling typical of Golden pothos), and green-Albino (sectorial leaves dominated by green and near-white chlorophyll-deficient patches). Three classes were derived from *Euphorbia pulcherrima* ornamental bracts: Albino (near-white to cream bracts with minimal visible chlorophyll), Green–Red (transitional material retaining green tissue while red pigmentation develops) and Red (full-pigmented red bracts). The remaining two classes corresponded to the green leaves of *Pelargonium zonale* (Geranium) and *Hibiscus rosa-sinensis* (Hibisco) (Table S1).

This composition intentionally mixed foliage and bract tissues because ornamental screening is usually performed on marketable organs. The geranium leaves were rounded to slightly lobed and lightly pubescent, the Hibiscus leaves were broader, smoother and glossy, the pothos leaves were entire and thick-textured, and the poinsettia classes were bract-based rather than leaf-based. The complete descriptor matrix is provided in Table S1. Each class contributed 100 independent plant-level samples: one healthy leaf or bract was randomly selected from each individual plant, and one spectrum was retained from that selected organ. Repeated readings from the same plant were not treated as additional biological replicates. Thus, the benchmark represents 900 individual plant-level spectra, not repeated spectra from a smaller set of plants. This balance was retained throughout the modelling workflow (see TOC) so that macro, weighted and balanced summaries could be interpreted directly without class-frequency correction.

For downstream analyses, the classes were also encoded to support compaction in the matrices, PCA plots and confusion charts. The benchmark was designed to be intentionally difficult: the strongest within-manifold crowding was expected among the pothos-derived green classes, whereas the poinsettia bract classes contributed more abrupt pigment-state contrasts across near-white, transitional green–red and fully red tissues.

### 2.2 Proximal Spectral Acquisition

Proximal reflectance measurements were obtained with a portable ASD Fieldspec 3 spectroradiometer (Malvern Panalytical/ASD Inc., Boulder, CO, USA), using a plant-probe, with nominal VIS–NIR–SWIR coverage from 350 to 2500 nm. For the present study, the analytical wavelength axis was standardized to 400–2400 nm at a spacing of 1 nm, yielding 2,001 spectral variables per sample. Measurements were collected under controlled conditions in a contact configuration and referenced to a calibrated white Spectralon standard and dark-current so that the exported values represented reflectance rather than raw signal intensity, which is consistent with common leaf-spectroscopy practices.^19^

The measured organs included leaves for geranium, green, green-Albino, Hibisco, lime and variegated, and ornamental bracts for albino, green–red and red plants. In mixed-sector classes, readings were taken from visually representative tissue regions so that the spectra reflected the intended phenotype rather than a spatial average across sharply contrasting sectors. To minimize artifacts, readings were taken from intact tissue, and care was taken to avoid damaged regions, strong shadows and obvious edge effects.

Instrument optimization and white-reference standardization were repeated as required during acquisition to maintain spectral consistency across the benchmark. Because the objective was phenotype discrimination rather than within-organ mapping, one representative reflectance curve was retained for each randomly selected plant organ. The resulting dataset therefore represents independent plant-level leaf- or bract-level proximal signatures rather than pixel-resolved hyperspectral images or repeated within-plant spectra; the overall analytical sequence is summarized in TOC. No hazardous chemicals, regulated biological agents, or destructive procedures were involved in spectral acquisition; standard instrument and plant-handling precautions were followed throughout.

#### 2.2.1 Spectral Export and Wavelength Domain Definition

The exported full-spectrum representation comprises 2,001 wavelengths spanning 400–2400 nm. Because spectral edges are more prone to noise and instrument-related instability, an additional quality-controlled representation was created by trimming 10 nm from each edge, yielding 1,979 wavelengths spanning 411–2389 nm. Both representations were retained for downstream sensitivity analysis.

A third representation was generated for reduced-band testing. Here, supervised feature ranking was performed within the training/validation development data after the plant-level split, and the selected wavelengths were then frozen before the independent test set was evaluated. This procedure identified 16 informative wavelengths after edge trimming, producing a compact configuration spanning 547–562 nm. This 16-band set was interpreted as an exploratory multispectral proxy rather than as a replacement for the full spectrum.

#### 2.2.2 Sample-Level Spectral Signature Generation

Each measurement was stored as a single reflectance vector paired with its class label. Unlike imaging-based workflows, no pixel aggregation or region-of-interest delineation was required because the pipeline directly produced one spectrum per sampled organ surface.

The resulting feature matrices had dimensions Xfull = 900 × 2001 for the full representation and Xtrim = 900 × 1979 for the edge-trimmed representation. The wavelength positions of the companion vectors were stored in nm, and each row was mapped to one of the nine ornamental classes (Table S1).

All downstream descriptive statistics, dimensionality reduction, feature ranking and predictive modelling were derived from these sample-level reflectance vectors.

### 2.3 Organization of the Spectral Dataset and Python Implementation

All post-acquisition processing was implemented in Python 3.13.9 within a single scripted pipeline to maximize transparency and reproducibility. Spectral organization, preprocessing, benchmarking and figure generation were performed programmatically from the same input workbook and split definitions.

### 2.4 Spectral Preprocessing

The primary preprocessing workflow applied Savitzky–Golay smoothing (window length = 11, polynomial order = 2) to the reflectance curves. Clipping to the physically plausible interval [0,1] was retained in the workflow used to generate the reported outputs. Polynomial baseline correction and standard normal variate (SNV) normalization were available as alternative options but were not enabled in the main benchmark.^21,33^

Accordingly, the three principal spectral representations considered here were (i) the full 400–2400 nm spectrum, (ii) the edge-trimmed 411–2389 nm spectrum, and (iii) the trimmed 16-band top-k configuration derived from ReliefF.^25^ This organization made it possible to evaluate how much performance could be retained after progressively stronger wavelength reduction.

Because the full and trimmed spectra preserve the natural ordering and redundancy of adjacent wavelengths, they were treated as the primary analytical representations, whereas the 16-band configuration was interpreted as a reduced-band sensitivity analysis.

### 2.5 Descriptive and Integrative Spectral Analyses

Descriptive analyses included classwise mean spectra, pairwise correlations among class means, one-versus-rest Pearson correlation profiles across wavelengths, coefficient-of-determination maps (*r* ^2^) and hierarchical clustering based on spectral distances between class means. These analyses were used to characterize the global similarity structure and to identify wavelength regions with class-specific leverage.^7–12,19,21,25,28–32^

For one-versus-rest association mapping, each class was encoded as a binary indicator (1 = focal class; 0 = remaining classes) so that the wavelength-wise correlations described class membership without imposing an ordinal structure on the labels.

For presentation, the signed Pearson coefficient was retained to show the direction of association, whereas *r* ^2^ was used to summarize attribution strength independently of sign. This dual representation allowed local class-specific contrasts to be compared with the broader multiclass ranking obtained from ReliefF.

### 2.6 Principal Component Analysis

Before PCA, each wavelength was standardized to zero mean and unit variance across samples so that broad high-intensity regions did not dominate the decomposition, and PCA was used strictly as an exploratory variance-partitioning tool.^34^

The PCA was interpreted jointly with the mean spectra and class neighbourhood structure to identify whether the dominant variance modes were concentrated in the visible range, the SWIR domain or both. Attention focused on the wavelength positions of the absolute loading maxima for PC1–PC3, as these peaks summarize the key spectral regions driving class separation.

In this context, PCA was used as an interpretative tool rather than as a classifier: its role was to summarize variance geometry and to anticipate where genuine spectral crowding might constrain supervised discrimination.

### 2.7 Feature Ranking and Selection via ReliefF

Supervised feature ranking was used to quantify which wavelengths contributed most strongly to multiclass separability and to define the 16-band reduced configuration used in the compact benchmark.^25^

$$w_{j}\leftarrow w_{j}-\frac{1}{\mathrm{mk}}\sum_{h\in H_{i}\;}\left|x_{\mathrm{ij}}-x_{\mathrm{hj}}\right|+\frac{1}{\mathrm{mk}}\sum_{m\in M_{i}\;}\left|x_{\mathrm{ij}}-x_{\mathrm{mj}}\right|$$

In practical terms, the ReliefF update increases a band’s weight when neighbouring samples from different classes diverge more strongly at that wavelength than neighbouring samples from the same class do. Bands with larger positive weights were therefore interpreted as more informative for class discrimination.

The resulting rank order was used descriptively to interpret wavelength importance and operationally to define the exploratory top-k representation. The ranking was not repeated separately inside each model family because the reduced-band experiment was designed to test one locked sensor-like configuration rather than to optimize a different wavelength subset for every classifier.

#### 2.7.1 Details and Hyperparameters of the ReliefF Implementation

ReliefF weighting was implemented in Python with internal min-max scaling to [0,1] before neighbour retrieval. Euclidean distance was used for the local neighbourhood comparisons; the neighbourhood size was fixed at 20, and 800 randomly sampled reference spectra were evaluated.^25^

Because the main objective of the reduced-band analysis was interpretative and deployment oriented, the final 16 wavelengths were selected from the edge-trimmed training/validation development set after the ranking had stabilized. These wavelengths were then reused across the compact classical benchmark, SHAP summaries and supplementary attribution analyses, while the independent test spectra remained unseen during wavelength selection and model optimization (Table S2).

### 2.8 Classification Modelling Framework

Ornamental identification was formulated as a balanced nine-class classification problem. A fixed stratified plant-level hold-out partition was used throughout: 35% of the samples were assigned to training (*n* = 315), 35% to validation (*n* = 315) and 30% to an independent test set (*n* = 270). Because one spectrum was retained per sampled plant organ, this partition separated individual plant-level spectra across training, validation and test subsets; each class contributed 35 samples to training, 35 to validation and 30 to testing. The test subset was locked before optimization and did not contribute to feature ranking, scaling-parameter estimation, hyperparameter screening or model fitting. Thus, the reported test scores represent plant-independent hold-out validation within the same acquisition campaign; cross-nursery, cross-operator, cross-season and cross-platform transfer to other sensing architectures are not reported here and are treated as deployment-scale future work.^1,4,14,19,30–32^

Model quality was assessed on the independent test set using overall accuracy, the error rate, macro-F1, weighted F1 and balanced accuracy. Confusion matrices were generated in raw counts and, where appropriate, in row-normalized form to reveal class-specific error structure.^35–38^

$$\mathrm{Accuracy}=\frac{1}{N}\sum_{i=1}^{N}oI\left(\hat{y_{i}\;}=y_{i}\right)$$

Errorrate=1−Accuracy

$$C_{\mathrm{ab}}=\sum_{i=1}^{N}oI\left(y_{i}=a\right)I\left(\hat{y_{i}\;}=b\right)$$

Training time, prediction throughput, total misclassification cost and model size were also retained to provide a deployment-oriented perspective on computational feasibility across the classical model families.

$${\mathrm{Precision}}_{c}=\frac{{\mathrm{TP}}_{c}}{{\mathrm{TP}}_{c}+{\mathrm{FP}}_{c}}$$

$${\mathrm{Recall}}_{c}=\frac{{\mathrm{TP}}_{c}}{{\mathrm{TP}}_{c}+{\mathrm{FN}}_{c}}$$

$$F1_{c}=\frac{2\;{\mathrm{Precision}}_{c}\;{\mathrm{Recall}}_{c}}{{\mathrm{Precision}}_{c}+{\mathrm{Recall}}_{c}}$$

$${\mathrm{Precision}}_{w}=\sum_{c}\frac{n_{c}}{N}\;{\mathrm{Precision}}_{c}$$

$${\mathrm{Recall}}_{w}=\sum_{c}\frac{n_{c}}{N}\;{\mathrm{Recall}}_{c}$$

$$F1_{w}=\sum_{c}\frac{n_{c}}{N}\;F1_{c}$$

In the sensitivity analyses, the best-performing model was reported for each spectral representation so that the consequences of wavelength trimming could be compared directly at the benchmark level rather than model by model.^25,28–32,35–38^

To address uncertainty in model ranking, a complementary paired validation analysis was added from the same workbook and split definitions. Independent-test accuracy, macro-F1 and balanced accuracy were summarized with 95% confidence intervals from 10,000 non-parametric bootstrap resamples of the test set, and paired classifier differences were evaluated with the exact McNemar test based on the same 270 held-out plant-level samples. These analyses were used to determine whether representation-level differences should be interpreted as statistically meaningful or only descriptive; the full results are reported in Tables S4–S5.^39–41^

### 2.9 Model Optimization Curves and Probabilistic Performance Summaries

Optimization-style diagnostics were generated for representative baseline workflows. For *k*-nearest neighbours, the number of neighbours was varied over a constrained grid using only the training/validation data; for shallow neural networks, discrete hidden-layer configurations were evaluated under the same principle. The independent test set was never used for model selection.

When a classifier returned class probabilities or continuous decision scores, one-versus-rest micro- and macro-averaged ROC-AUC and average-precision summaries were computed to characterize the ranking quality beyond hard class assignment.^35,37,38^

$$\mathrm{TPR}=\frac{\mathrm{TP}}{\mathrm{TP}+\mathrm{FN}}$$

$$\mathrm{FPR}=\frac{\mathrm{FP}}{\mathrm{FP}+\mathrm{TN}}$$

$$\mathrm{Precision}=\frac{\mathrm{TP}}{\mathrm{TP}+\mathrm{FP}}$$

$$\mathrm{Recall}=\frac{\mathrm{TP}}{\mathrm{TP}+\mathrm{FN}}$$

These probabilistic summaries were especially useful for determining whether models that made similar numbers of hard errors nevertheless differed in their confidence in the ordering of the nine ornamental classes.

### 2.10 Deep Learning Models for 1D Spectral Signatures

Deep learning was applied directly to the one-dimensional reflectance sequences, treating each sample as an ordered vector of length 2,001. The same 35:35:30 stratified split used in the classical benchmark was retained so that the reported scores could be compared across modelling paradigms.

The evaluated architectures were MLP_1D, MLP_Wide, MLP_Deep, CNN1D, CNN1D_Deep, ResNet1D, Inception1D, TCN1D, BiLSTM1D and Transformer1D. All the networks were trained with the Adam optimizer (learning rate = 1 × 10–3), a batch size = 30 and a maximum of 25 epochs, and early stopping (patience = 6) to restore the weights associated with the lowest validation loss.^26,42–50,52^

Training therefore emphasized comparable optimization budgets rather than architecture-specific tuning, allowing the ranking of the deep models to be interpreted primarily as a function of representational fit to the spectral data.

$$L=-\frac{1}{N}\sum_{i=1}^{N}\log\left(p_{i,y_{i}}\right)$$

Independent-test performance was summarized using accuracy, macro-F1, precision, recall, number of trainable parameters and epochs completed. Training and validation histories were also retained to determine convergence stability and overfitting.^35–41,48,49^

### 2.11 Reproducibility and Figure Generation

All figures, tables and supplementary outputs were generated programmatically from the same analysis pipeline used for preprocessing, feature ranking and model fitting. This included the mean spectra, PCA projections, wavelength-importance profiles, benchmark panels, confusion matrices and deep-learning learning curves for analysis in plants.^50–57^

The analytical environment included Python 3.13.9 together with NumPy 2.2.6, Pandas 2.3.3, Matplotlib 3.10.5, Seaborn 0.13.2, SciPy 1.16.3, scikit-learn 1.7.2, OpenPyXL 3.1.5 and PyTorch 2.2.2. Computational routines were executed on a workstation equipped with an NVIDIA GeForce RTX 4090 GPU, an Intel Core i9-14900HX CPU, 128 GB of RAM and NVMe storage, which was sufficient for repeated full-spectrum benchmarking and deep-learning training without additional data reduction.

## 3 Results

### 3.1 Benchmark Composition and Ornamental Phenotypes

The benchmark comprised 900 proximal reflectance measurements distributed evenly across nine ornamental classes (100 samples per class; Figure 1; Table S1). Four classes represented *E. aureum* foliage phenotypes (green, green-Albino, lime, and variegated), three represented *E. pulcherrima* bract phenotypes (Albino, green–red, and red), and the remaining two represented leaf classes from *P. zonale* (Geranium) and *H. rosa-sinensis* (Hibisco). The dataset therefore combined dark-green, lime-green, cream/white-sectorial, green–yellow-cream marbled, transitional green–red and fully red tissues within a single balanced benchmark. This deliberately broadened the visible phenotype space beyond simple species discrimination and created a demanding scenario in which some classes differed mainly in terms of pigment distribution within related ornamental material.

**Figure 1:**
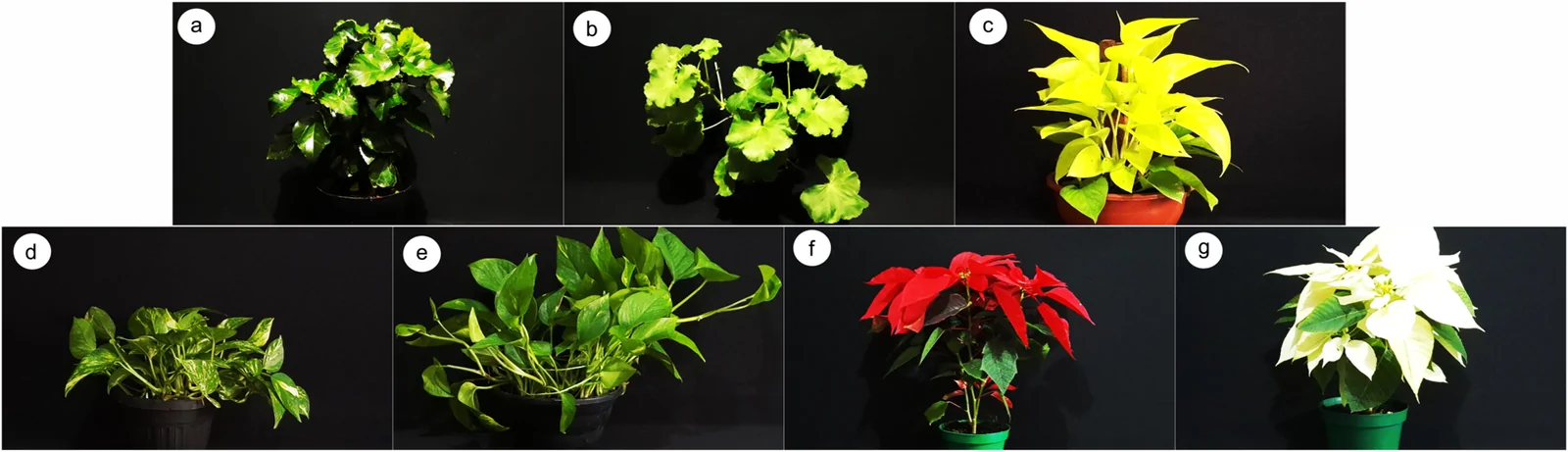
Representative ornamental material used in the proximal spectroscopy benchmark. The panels illustrate representative plants and the characteristic organs measured for the corresponding classes: (a) Hibiscus leaves (Hibisco class); (b) Pelargonium leaves (Geranium class); (c) lime-green pothos leaves (Lime class); (d) green-yellow-cream marbled pothos leaves (Variegated class); (e) uniform dark-green pothos leaves (Green class); (f) fully red poinsettia bracts (Red class); and (g) near-white/cream poinsettia bracts (Albino class). The figure provides a visual reference for the class descriptors in Table S1 and clarifies why the benchmark combines species-level leaf classes with phenotype-level leaf and bract states..

The phenotypes were defined from the sampled organ itself: bracts for the three poinsettia classes and leaves for all remaining classes. Representative examples of the measured material are shown in Figure 1.

The full analytical sequence, from tissue acquisition to classification benchmarking, is summarized in Figure 2.

**Figure 2:**
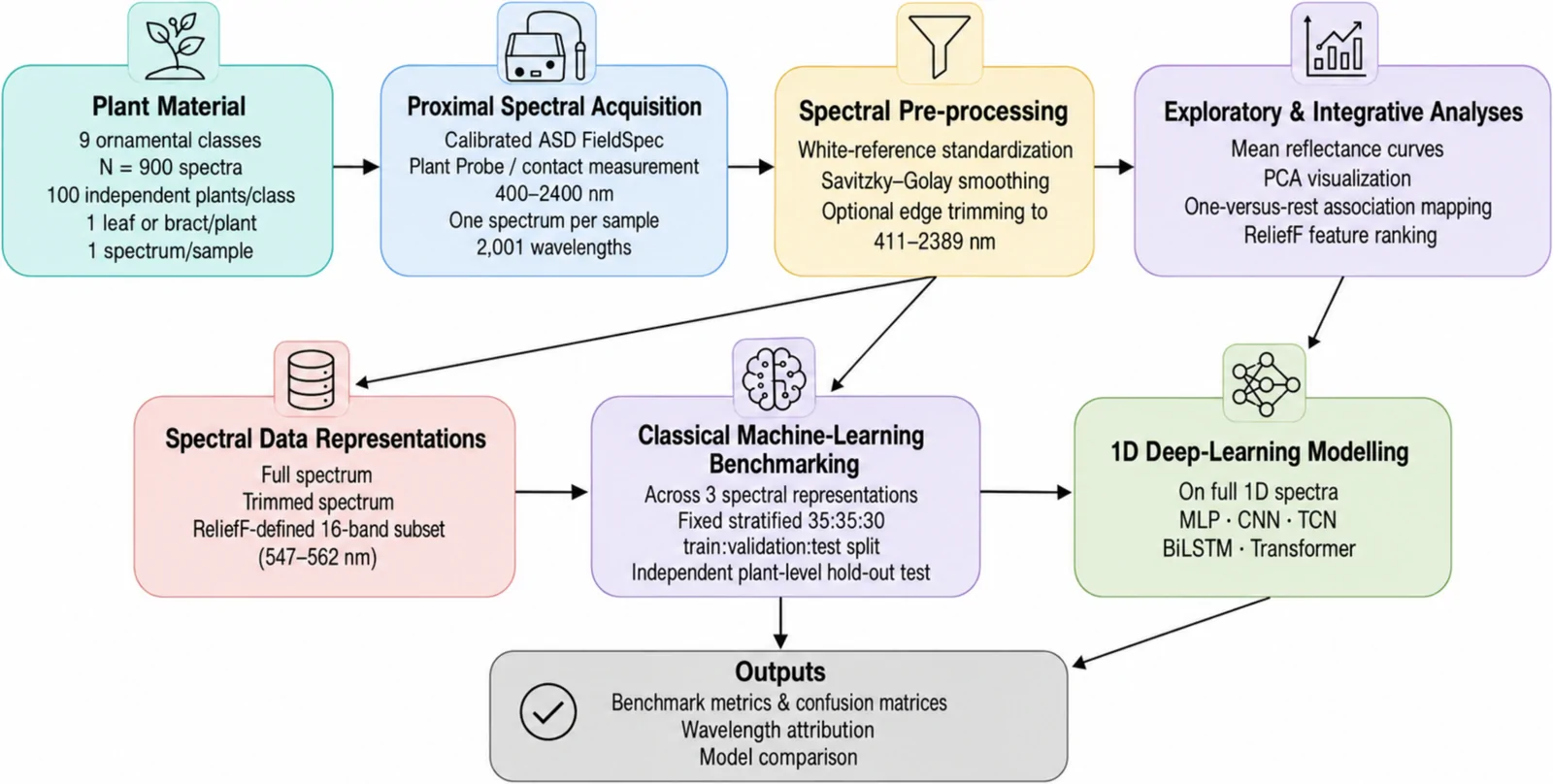
Analytical workflow for ornamental plant identification using proximal VIS–NIR–SWIR reflectance spectroscopy. The workflow summarizes plant-level sampling, calibrated contact spectral acquisition, white-reference standardization, Savitzky–Golay smoothing, optional edge trimming, exploratory spectral analyses, ReliefF-based band ranking, classical machine-learning benchmarking, and 1D deep-learning modelling. Three spectral representations were evaluated: the full 400–2400 nm spectrum, the edge-trimmed 411–2389 nm spectrum, and the ReliefF-defined 16-band subset at 547–562 nm. Model performance was assessed using a fixed stratified 35:35:30 train/validation/test split, with independent plant-level test spectra withheld from training and tuning.

### 3.2 Class-Level Reflectance Organization across the VIS–NIR–SWIR Domain

The class-averaged reflectance curves were strongly structured across the full 400–2400 nm range (Figure 3a). Separation was already visible in the visible domain, where Albino and Lime showed high reflectance, Red and Green–Red exhibited distinctive pigment-driven behaviour, and the green-derived classes occupied a more crowded central manifold. Beyond the visible range, the NIR and SWIR domains retained clear class-dependent differences, indicating that discrimination was not driven by color alone but also by broader water- and structure-sensitive reflectance behaviour.

**Figure 3:**
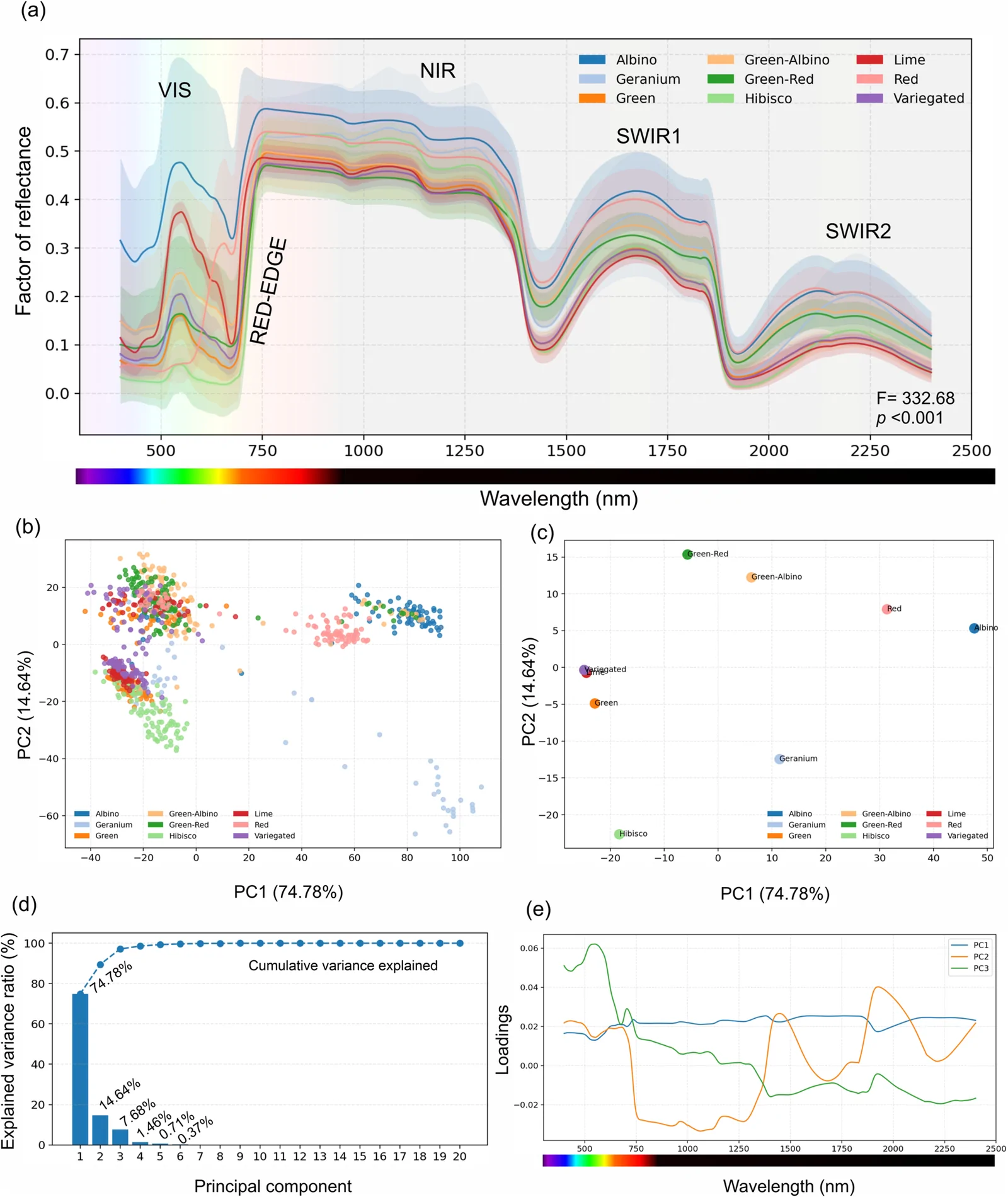
Class-level reflectance organization and PCA-derived variance structure for the nine ornamental classes. (a) Mean reflectance curves across the 400–2400 nm domain, showing the high-reflectance albino/lime tissues, the crowded green-centred classes and the distinctive red-bract behaviour. (b) PCA scores for all samples projected onto PC1 and PC2. (c) The same PC space summarized as class centroids. (d) Explained variance of the first 20 principal components with cumulative variance overlaid. (e) Loading profiles for PC1–PC3, highlighting the visible and SWIR regions that contributed most strongly to the dominant variance modes.

The correlation structure among the class means confirmed this organization. The most similar class pairs were green versus variegated (*r* = 0.997), green-Albino versus variegated (*r* = 0.996) and geranium versus green (*r* = 0.993), whereas the least similar relationships included Albino versus red (*r* = 0.815), lime versus red (*r* = 0.838) and Albino versus Hibisco (*r* = 0.850). In other words, the main classification difficulty was concentrated among the green-centred classes, while the red and high-albedo phenotypes occupied more distant spectral regions.

### 3.3 PCA Reveals a Strongly Compressed Low-Dimensional Structure

Principal component analysis revealed that the ornamental spectra lay on a highly compressed low-dimensional manifold. PC1 explained 74.78% of the variance, PC2 explained 14.64%, and PC3 explained 7.68%; thus, the first three components together accounted for 97.11% of the total spectral variation (Figure 3b–e). These results indicate strong wavelength redundancy and suggest that the dominant class differences are governed by broad, physically coherent spectral regimes rather than by isolated noisy bands. The loading maxima further showed that the leading axes were drawn in both the visible and SWIR regions: absolute PC1 peaks were concentrated at approximately 1377–1380 and 1597–1602 nm, PC2 peaked at approximately 1922–1931 nm, and PC3 peaked at approximately 543–552 nm.

### 3.4 Wavelength-Resolved Associations and Supervised Ranking Highlights a Compact Green Window

One-versus-rest association mapping confirmed that no single wavelength explained every class equally well (Figure 4a–b). Instead, each ornamental class exhibited its own spectral emphasis. Albino had the strongest positive association at 400 nm (*r* = 0.602; *r*2 = 0.362), Hibisco had the strongest negative association at 715 nm (*r* = −0.433; *r*2 = 0.188), and Red had a strong positive maximum at 2022 nm (*r* = 0.434; *r*2 = 0.188). These maxima demonstrate that the discriminative signal is distributed across both the visible and the SWIR regions, align hierarchical cluster (Figure 4c) depending on the class under consideration (Figure 4).

**Figure 4:**
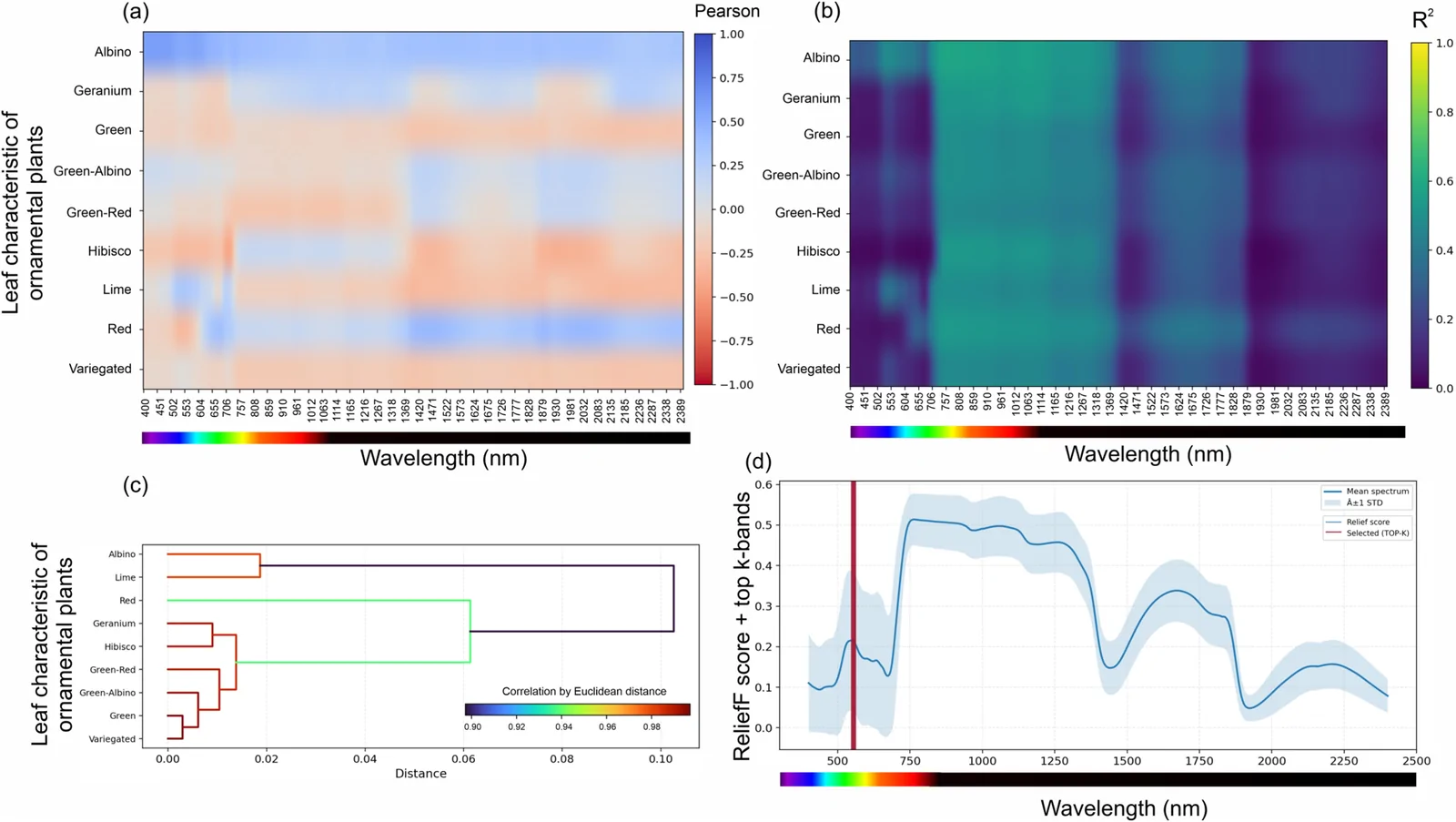
Wavelength-resolved association structure, class similarity and supervised band ranking for the ornamental benchmark. (a) One-versus-rest Pearson correlations (*r*) between reflectance and class membership across wavelengths. (b) The same map expressed as *r* ^2^. (c) Hierarchical clustering of class-mean spectra based on spectral distance. (d) ReliefF importance profile across wavelengths, displayed together with the mean spectrum and markers for the 16 selected bands.

The multiclass ranking nevertheless converged on a compact visible interval when ReliefF was used to identify the most reusable bands. The selected 16-band configuration spanned 547–562 nm and peaked at 553 nm, whereas the strongest ANOVA effect size was located at 1448 nm (eta2 = 0.633) outside the reduced set (Figure 4d; Figure S2; Table S2). This divergence is important: the green window is highly efficient for compact multiclass ranking, but the broader spectral space still contains complementary information, especially in the SWIR band. The hierarchical clustering of class-mean spectra mirrored the correlation analysis by grouping the green-centred phenotypes together and placing the more distinctive red, lime and albino classes on more distant branches.

A representation-level summary of the sensitivity analysis is given in Table 1.

**Table 1:** Sensitivity Summary across Spectral Representations Evaluated on the Independent Test Set^a^

Representation	Wavelength Coverage (nm)	Bands	Best Model	Macro-F1 (%)	Balanced Accuracy (%)
Full spectrum	400–2400	2001	Wide neural network	82.56	82.59
Trimmed spectrum	411–2389	1979	Linear SVM	81.99	81.85
Trimmed spectrum + top-k	547–562	16	Linear discriminant	60.77	60.74

a For each representation, the table shows the wavelength coverage after preprocessing, number of retained bands, best-performing model, macro-F1 score and balanced accuracy. The final row corresponds to the ReliefF-selected 16-band configuration derived from the trimmed spectrum and should be interpreted as an exploratory reduced-band benchmark rather than as a replacement for full spectral coverage.

### 3.5 Spectral Representation Sensitivity and Reduced-Band Classical Benchmarking

Sensitivity analysis across spectral representations revealed that most of the predictive power was retained when the wavelength axis remained broad (Table 1). Using the full 400–2400 nm spectrum, the strongest model was a wide neural network with a macro-F1 = 82.56% and a balanced accuracy = 82.59%. The edge-trimmed 411–2389 nm representation performed almost identically, with the linear SVM reaching a macro-F1 = 81.99% and a balanced accuracy = 81.85%. In contrast, compressing the problem to the 16 ReliefF-selected wavelengths reduced the best macro-F1 to 60.77% and the balanced accuracy to 60.74%, showing that the compact green window is informative but not sufficient to preserve the full class structure (Figure 5a–e).

**Figure 5:**
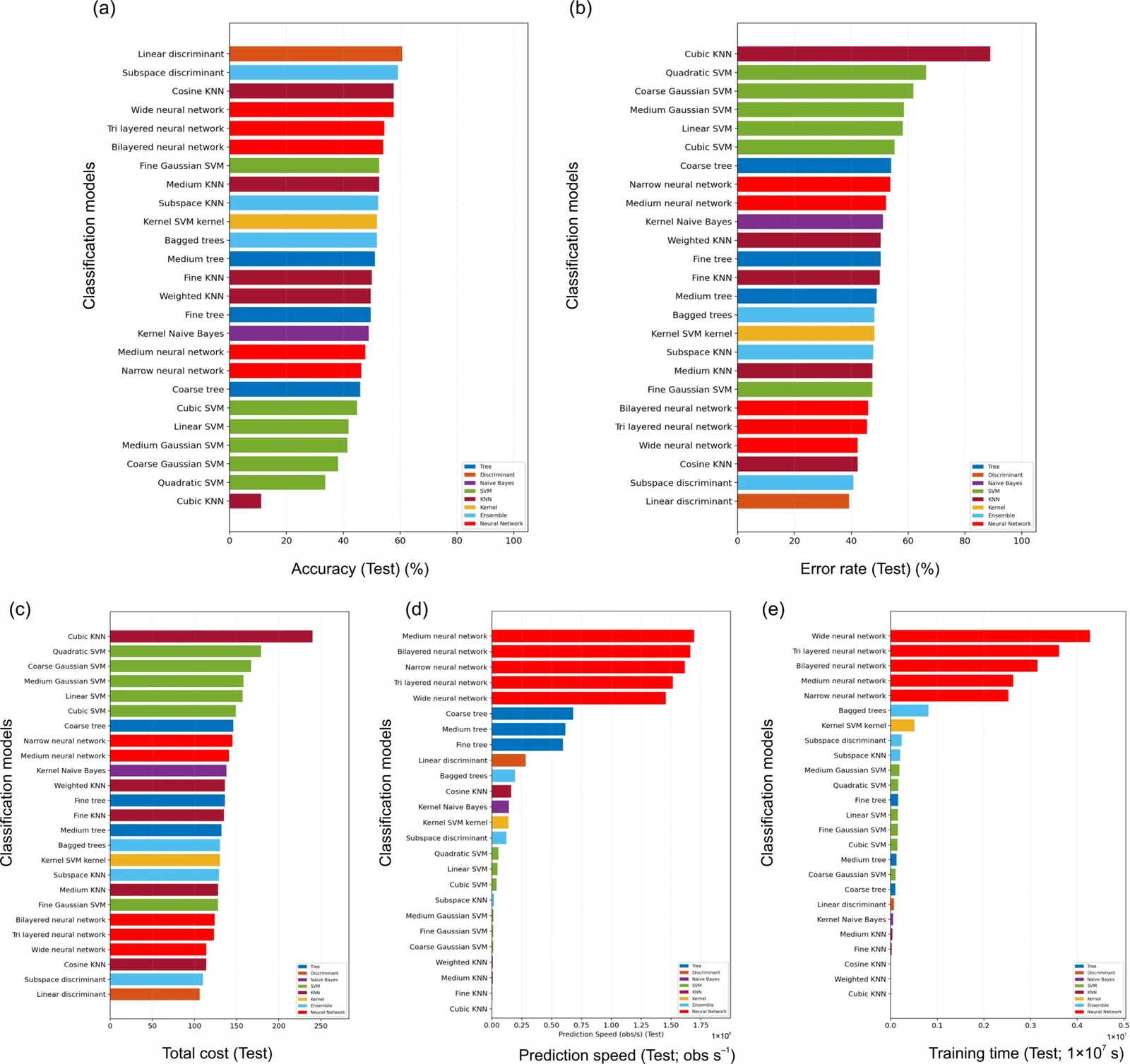
Classification benchmarking and computational profiling for the reduced 16-band ornamental benchmark. Panels show the independent-test (a) accuracy, (b) error rate, (c) total misclassification cost, (d) prediction throughput and (e) training time across the evaluated classical models. All the models were fitted under the same fixed split, so the figure visualizes the performance-efficiency trade-off after aggressive wavelength compression to the 547–562 nm window.

At the model-by-model level, this representation effect was systematic rather than restricted to the single best classifier (Figure 6). Among the 24 comparable paired model contrasts, the median absolute macro-F1 difference between full and edge-trimmed spectra was only 0.35 percentage points, and 22 contrasts differed by less than 2 percentage points. In contrast, the 16-band green window representation reduced macro-F1 relative to its better broad-spectrum counterpart in 23 of those 24 contrasts, with a median penalty of 26.84 percentage points. Thus, the compact-score loss was not a model-selection artifact; it reflected a representation bottleneck caused by removing the complementary spectral context needed by most classifier families.

**Figure 6:**
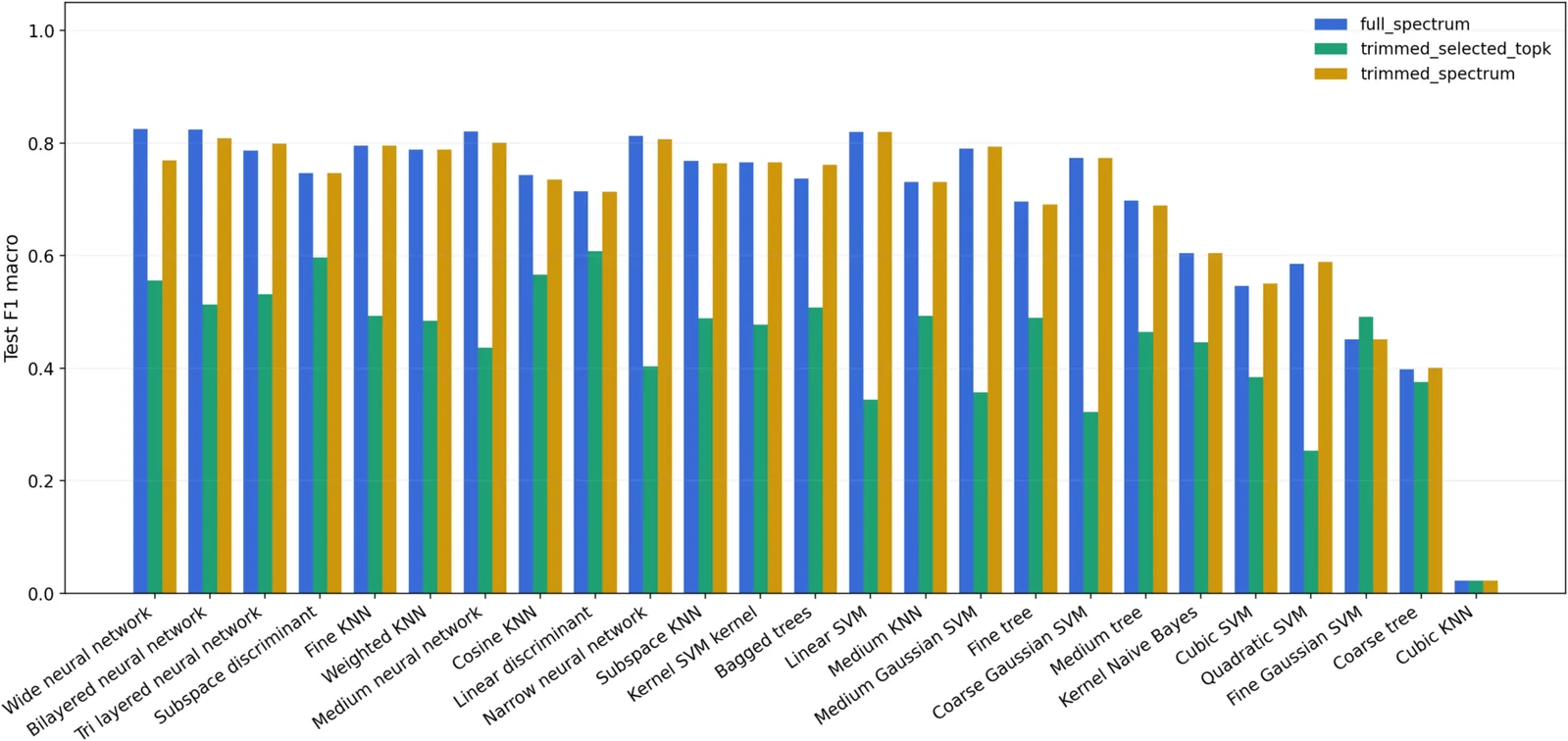
Analysis of classifier performance across spectral representations. Independent-test macro-F1 obtained for each evaluated classical machine-learning model using the full spectrum (400–2400 nm), the edge-trimmed spectrum (411–2389 nm), and the ReliefF-defined reduced 16-band subset (547–562 nm). Overall, the full and edge-trimmed spectra yielded similar performance across most models, whereas the reduced green-window subset generally decreased classification fidelity, indicating that these selected bands are informative but do not replace broader VIS–NIR–SWIR spectral coverage.

The added uncertainty analysis supported this interpretation without overclaiming small model-rank differences (Tables S4–S5). In the paired representation-level check, the full-spectrum and edge-trimmed linear SVM results were nearly indistinguishable for the held-out plants (macro-F1 = 82.63% and 81.90%, respectively; exact McNemar *P* = 0.500). In contrast, both broad-spectrum representations outperformed the 16-band green-window LDA model (macro-F1 = 60.77%; exact McNemar *P* < 0.001 for both paired comparisons). Thus, the statistical evidence supports the broad-versus-compact representation contrast, whereas the small difference between the two broad-spectrum settings should be interpreted descriptively rather than as a significant superiority claim.

Within this reduced 16-band benchmark, linear discriminant analysis was the strongest classical model, followed by subspace discriminant and cosine KNN (Figure 5; Table 2). The leading compact models therefore favored simple linear or distance-based discrimination rather than high-capacity nonlinear boundaries. At the class level, the linear discriminant model retained particularly strong recall for Hibisco (93.33%), Lime (86.67%) and Red (70.00%), whereas the recall for Green-Albino (30.00%) and Green–Red (33.33%) remained more difficult.

**Table 2:** Top-Performing Models in the Trimmed 16-Band Benchmark, Ranked by the Independent-Test Macro-F1 Score^a^

Rank	Model	Group	Macro-F1 (%)	Balanced Accuracy (%)
1	Linear discriminant	Discriminant	60.77	60.74
2	Subspace discriminant	Ensemble	59.68	59.26
3	Cosine KNN	KNN	56.64	57.78
4	Wide neural network	Neural network	55.60	57.78
5	Tri layered neural network	Neural network	53.16	54.44
6	Bilayered neural network	Neural network	51.35	54.07
7	Bagged trees	Ensemble	50.77	51.85
8	Fine KNN	KNN	49.32	50.00

a The classifier family, macro-F1 score and balanced accuracy are reported for the eight strongest compact models obtained after aggressive wavelength reduction.

The confusion matrices were largely structured rather than random (Figure S7). For the best compact model, the greatest confusion was that of Green–Red → Hibisco (10/30; 33.3%), Green-Albino → Green (9/30; 30.0%), Geranium → Green (9/30; 30.0%), Variegated → Green (8/30; 26.7%), Green → Variegated (7/30; 23.3%) and Albino → Green (7/30; 23.3%). These errors reinforce the descriptive analyses: the densest confusion occurred among green-centred classes with partially overlapping reflectance behaviour, whereas Lime, Hibisco and Red were relatively stable.

Figure 6 and Figure S7 should therefore be read together: Figure 6 quantifies the cross-model effect of spectral compression, whereas Figure S7 localizes the biological consequence of that compression to specific off-diagonal errors. The compact green-window model still preserved strongly separable classes such as Hibisco, Lime and Red, but it lost resolution among intermediate, transitional and green-centred phenotypes. This pattern supports the conclusion that a reduced ornamental sensor should not be designed as a single contiguous green block, even when that block is highly ranked by ReliefF.

The independent test results for all the one-dimensional deep learning architectures are summarized in Table 3.

**Table 3:** Independent Test-Set Performance of the One-Dimensional Deep Learning Architectures Trained on the Full 2,001-Band Reflectance Curves^a^

Model	Accuracy (%)	F1 Score (%)	Precision (%)	Recall (%)	Parameters	Epochs
MLP_Deep	80.74	80.99	83.45	80.74	554,249	25
MLP_1D	80.74	80.77	82.19	80.74	265,097	25
MLP_Wide	80.00	80.16	81.60	80.00	1,29,641	18
Transformer1D	64.07	62.82	64.56	64.07	67,657	25
ResNet1D	55.56	56.10	58.49	55.56	76,105	25
BiLSTM1D	54.07	53.77	56.02	54.07	35,465	25
Inception1D	53.70	52.25	55.03	53.70	18,009	25
CNN1D_Deep	51.11	47.32	51.57	51.11	54,569	25
CNN1D	47.04	47.44	53.38	47.04	11,337	25
TCN1D	44.44	42.71	44.36	44.44	75,849	25

a The accuracy, F1 score, precision and recall are reported as percentages; the parameter count indicates the number of trainable weights, and the number of epochs indicates the number of completed training epochs under the shared training budget.

### 3.6 Deep Learning on Full One-Dimensional Spectra

Deep learning on the full 2,001-band spectra produced a ranking distinct from that of the reduced-band classical benchmark (Table 3; Figures 7–8). The strongest full-spectrum deep model according to the F1 score was MLP_Deep, which achieved 80.74% accuracy, 80.99% F1, 83.45% precision and 554,249 trainable parameters. MLP_1D matched MLP_Deep in terms of accuracy (80.74%) and followed closely in terms of the F1 score (80.77%), whereas MLP_Wide achieved 80.00% accuracy. Transformer1D formed an intermediate tier (64.07%), and the convolutional, recurrent and temporal models remained lower under the shared training budget. The training trajectories and a representative full-spectrum confusion matrix are shown in Figure 7.

**Figure 7:**
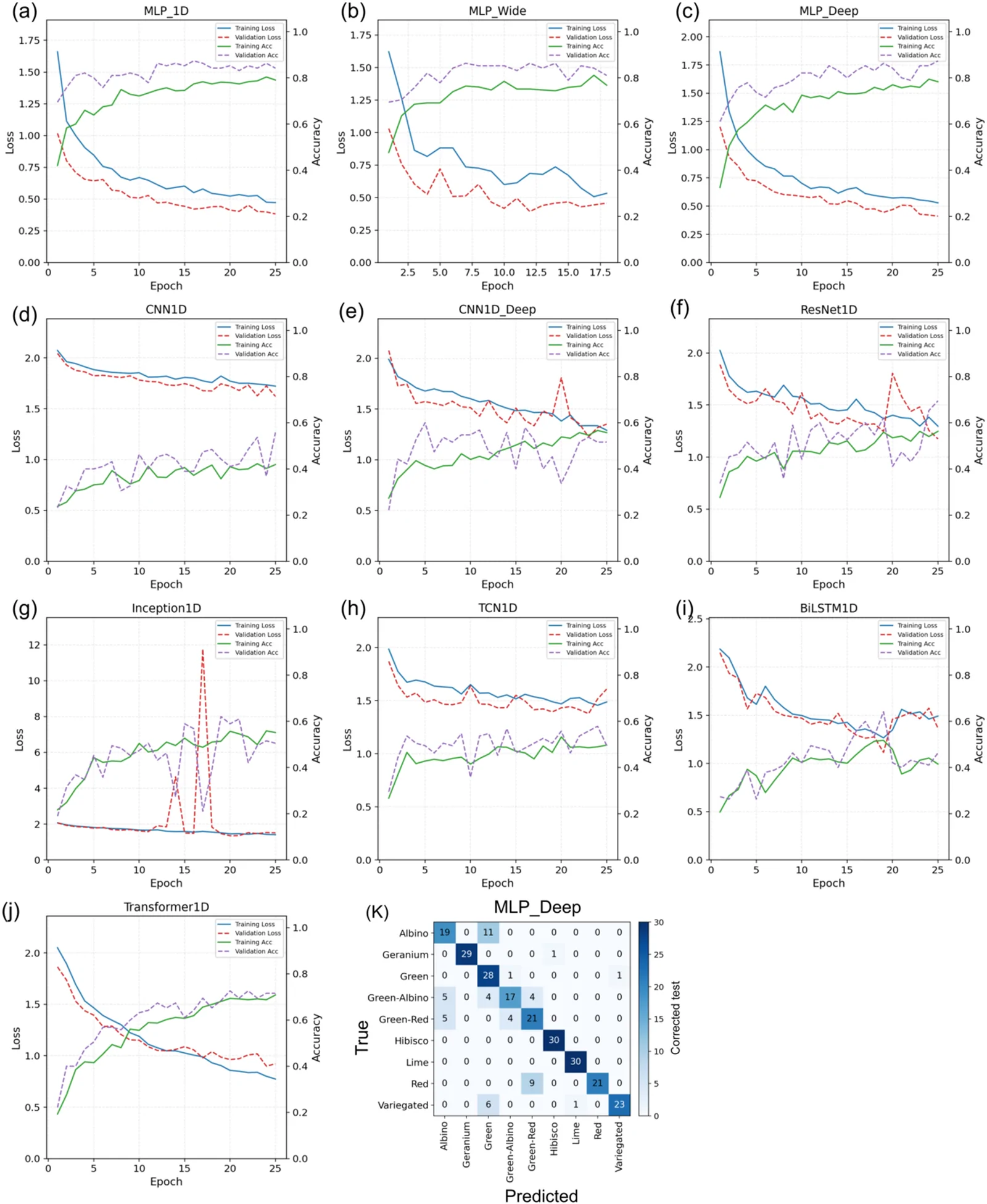
Training dynamics of the one-dimensional deep learning architectures fitted to the full ornamental spectra. Panels (a–j) show epoch wise training and validation loss and accuracy traces for the evaluated models under the shared optimization budget. Panel (k) shows the independent-test confusion matrix for a top-performing MLP-type full-spectrum model (30 test samples per class), illustrating the residual class-specific error structure after full-spectrum training.

**Figure 8:**
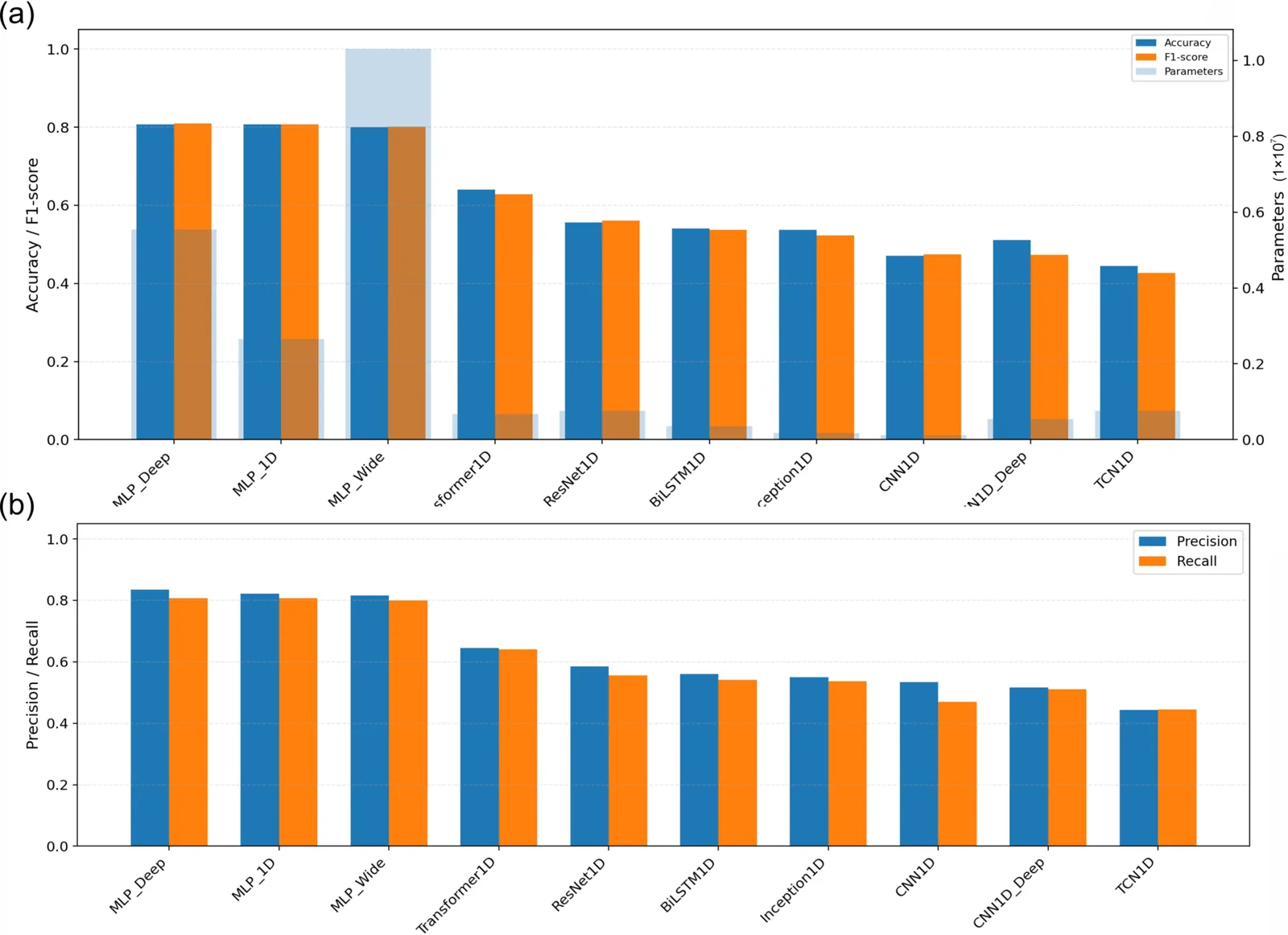
Summary of independent-test performance and model capacity across the deep-learning architectures trained on the full ornamental spectra. (a) Accuracy and F1 score are plotted together with the number of trainable parameters, highlighting the relationship between the predictive performance and model size. (b) Precision and recall are shown for the same architectures to compare error symmetry across models.

The optimization traces reinforced this interpretation. The MLP family converged rapidly and showed relatively stable validation behaviour, whereas the more structured sequence architectures displayed noisier trajectories and weaker generalization despite comparable optimization budgets. In the confusion matrix of the leading MLP family, Hibisco and Lime were recognized almost perfectly, while the main residual overlap persisted among Green, Green-Albino, Variegated and Green–Red. The full-spectrum deep models therefore recovered substantially more of the multiclass structure than the compact 16-band classical benchmark did, yet they still did not exceed the best full-spectrum sensitivity result reported in Table 1. A cross-architecture summary of the predictive performance and model size is shown in Figure 8.

## 4 Discussion

### 4.1 Spectral Contrasts across Ornamental Classes are Optically Plausible

The spectral organization observed here is consistent with what would be expected from ornamental tissues defined by visible phenotype, organ identity and taxonomic source. The benchmark did not compare nine arbitrary colours: it combined four pothos-derived foliage phenotypes, three poinsettia bract phenotypes, one geranium leaf class and one hibiscus leaf class. High-albedo cream/near-white tissues, red bracts and green foliage are expected to differ in visible absorption and scattering, and the SWIR domain is expected to add sensitivity to water-related and structural optical behaviour. However, pigment concentrations, water content and anatomical traits were not measured directly in this study; therefore, mechanistic explanations are presented as literature-supported optical interpretations rather than as direct biochemical or anatomical validation.^7–12,58,59^

The PCA loadings strengthen that interpretation. PC1 peaked at approximately 1377–1380 and 1597–1602 nm, PC2 at approximately 1922–1931 nm, and PC3 at approximately 543–552 nm; thus, the leading axes combined classic water- and structure-sensitive SWIR behaviour with a narrow window of visible green. In biological terms, the visible contribution is consistent with the differences among chlorophyll-rich Jade-type foliage, chlorophyll-poorer Neon-type foliage, sectorial Golden-type pothos leaves, glossy Hibiscus leaves, lightly pubescent geranium leaves, and chlorophyll-poor versus anthocyanin poinsettia bracts. This alignment between unsupervised variance geometry and physically interpretable spectral regions precisely explains the explanatory power of proximal spectroradiometry in plant phenotyping.^7–12,58–60^

Class origin therefore mattered. The strongest leverage came from comparisons that crossed both pigment state and organ type, especially between chlorophyll-poor or anthocyanin poinsettia bracts and chlorophyll-rich leaves, whereas the crowded region of the benchmark was dominated by foliage classes that shared broadly similar mesophyll organization but differed mainly in sectoring or chlorophyll abundance.

This design also means that the classifier cannot be used to partition the independent contribution of species, organ type and colour phenotype. This separation would require a factorial experiment within a single species, organ and developmental stage.^13–19,58,59^ The present objective differed: To test whether the marketable leaf and bract classes encountered in ornamental screening can be identified from plant-level proximal reflectance spectra.^1–3,6,13–19^

### 4.2 Low-Dimensional Structure Explains Both Success and Failure Modes

The first three principal components explained 97.11% of the total variance, which is high for a nine-class biological dataset and helps explain why robust discrimination was possible.^13–16,19,21,34,61^ Adjacent wavelengths in plant spectra are strongly collinear; thus, many apparently high-dimensional problems are in practice driven by a small set of smooth spectral modes. Under those conditions, simple models remain competitive because they operate on a compressed, redundant geometry rather than on a truly unconstrained feature space.^13–16,19,21,34,61^

Moreover, the same low-dimensional structure explains the remaining errors.^13–16,21,34,61^ The most similar class pairs—Green, Variegated, Green-Albino and Geranium—occupied crowded neighbourhoods in the correlation structure and then dominated the confusion matrices.^16,34,61,62^ Three of these four classes were pothos-derived foliage states, so the classifier often had to separate pigment distributions within closely related leaf architecture rather than distinct organ types.^58,60,62^ That agreement between unsupervised geometry and supervised error structure is a useful diagnostic because it suggests that residual misclassification reflects genuine spectral overlap between related ornamental phenotypes, not merely optimization instability.^27,61,62^

This point is especially important for interpreting the green–red class. Transitional poinsettia material retained green tissue while red pigmentation developed; thus, it occupied an intermediate spectral position rather than behaving like a fully distinct red class. In practical terms, this is exactly the kind of ambiguity that a nursery-grade classifier must handle.^58^

### 4.3 *Full-Spectrum Retention* versus *Compact Sensor Design*

A key practical outcome of this study is the explicit contrast between the broad-spectrum and 16-band representations. Trimming the unstable edges had almost no penalty, which implies that a well-conditioned 411–2389 nm representation captures nearly all of the useful information for morphological trait in plants.^21,28–32^ However, restricting the data to the 16 ReliefF-selected wavelengths reduced the best benchmark score by more than 20 percentage points.^25,28–32,58^ Optically, this compact window is plausible because it samples the green plateau where dark-green, lime and sectorial pothos states diverge, but it misses part of the red absorption, red-edge shape and SWIR information that helps resolve poinsettia bracts, Hibiscus leaves and the most confusable green phenotypes. The green window is therefore a highly informative local cue, but it is not a complete surrogate for the broader VIS–NIR–SWIR signal.^7–12,58,60^

Importantly, the contrast between ReliefF and ANOVA is itself informative. ReliefF was concentrated in the 547–562 nm interval because that region is efficient for local multiclass discrimination, whereas ANOVA peaked at 1448 nm, indicating that a strong global between-class structure also exists outside the selected window. ReliefF also has a practical limitation in hyperspectral data: adjacent wavelengths are highly correlated, so a local neighbour-based ranking can select a compact cluster of redundant bands even when complementary information is distributed elsewhere. The operational implication is that reduced-band sensor design for ornamental screening should probably follow a distributed strategy—anchoring a small number of green bands while retaining complementary red-edge and NIR/SWIR coverage-rather than collapsing the system to a single narrow visible interval.^25,30,31^

The sensitivity panel and the compact confusion matrices also clarify the practical failure mode of the 16-band configuration. The score drop in Figure 6 was not concentrated in one classifier family; it appeared across SVMs, KNNs, neural networks and ensemble/discriminant models, indicating a representation-level bottleneck rather than a single-algorithm limitation. Figure S7 then shows how that bottleneck is expressed: off-diagonal mass accumulated mainly among the green, variegated, green-albino, geranium and green–red classes, while the more distinctive Hibisco, lime and red classes remained relatively stable. Operationally, this means that reduced “hardware” should retain distributed spectral cues capable of resolving the crowded green manifold, not only wavelengths that separate visually high-contrast phenotypes.

The reduced-band results also reveal why linear discriminant analysis rose to the top of the compact benchmark. Once the signal is compressed into a very narrow and highly correlated visible interval, linear class boundaries appear sufficient to exploit the remaining between-class differences. In that regime, extra model flexibility provides little benefit and can even magnify instability.^63^

From a translational perspective, this is encouraging. This suggests that simplified multispectral hardware could support useful preliminary sorting or screening of ornamental material, but it also warns against the assumption that the wavelengths most frequently highlighted by feature ranking are enough to preserve full classification fidelity on its own.

### 4.4 Representation Mattered More than Architectural Sophistication

The full-spectrum deep-learning benchmark reinforces the idea that representation mattered more than architectural sophistication did in this dataset. MLP_Deep and MLP_1D performed best, whereas the more structured convolutional, recurrent and transformer-style models did not close the gap further. This outcome is reasonable rather than contradictory: the input spectra are smooth, strongly collinear one-dimensional sequences; the sample size is modest relative to the flexibility of modern sequence architectures; and all architectures were trained under a shared optimization budget rather than under architecture-specific tuning. Under those conditions, a well-regularized multilayer perceptron or a linear-margin model can match the statistical structure of the data more effectively than models designed for large datasets with richer local motifs or long-range sequence dependencies.^27,42–50,64^

The same logic explains why the wide neural network was the best model in terms of the full-spectrum sensitivity summary, whereas the linear SVM remained competitive on the trimmed representation. Broad spectral coverage preserved enough complementary information for moderately flexible models to benefit, but the data still did not support a clear advantage for the most elaborate sequence-oriented networks. In other words, increasing model complexity was less important than preserving the wavelength regions that carried distinct pigment and SWIR cues.

For deployment, this is a useful outcome: MLP-type models are relatively easy to retrain, audit and export, whereas the heavier sequence architectures would only be justified if substantially larger ornamental datasets revealed a clearer advantage.

### 4.5 Implications for Ornamental Horticulture and Proximal Phenotyping

Although the present benchmark was constructed under controlled conditions, the results are directly relevant for ornamental horticulture. Non-destructive reflectance classification could support nursery sorting, cultivar or phenotype verification, quality control of color states, and rapid phenotyping during breeding or stress screening workflows. Ornamentals are particularly suitable for this type of sensing because their commercial value is often tied directly to visible pigmentation patterns that are simultaneously biologically meaningful and optically measurable.^2,3,6,58^

Equally important, the workflow used here is operationally transparent. The class averages, PCA geometry, wavelength importance profiles and confusion patterns all tell a coherent story, which makes the system easier to audit than an accuracy-only black box does. For applications in nurseries and breeding pipelines, interpretability is valuable because it helps distinguish genuine phenotype-linked signals from transient acquisition artifacts.

At a practical level, the current benchmark suggests three immediate use cases: verification of dark-green versus lime pothos lines, confirmation of sectorial variegation classes before market or propagation, and discrimination of poinsettia material across near-white, transitional green–red and fully red bract states.

### 4.6 Limitations, Future Directions, and Overall Implications

The present benchmark has specific boundary conditions that should be interpreted in relation to its classification objective. The independent test set consisted of plant-level spectra withheld from model fitting and model selection; therefore, the reported test metrics quantify generalization to previously unseen plants within the same acquisition domain, not resubstitution performance. This is an appropriate independent hold-out validation for the present balanced benchmark, although it is not intended to claim cross-nursery or cross-platform deployment without additional transfer experiments.^1,4,14,19,30–32,62^

The spectra were acquired with a calibrated high-resolution ASD FieldSpec contact configuration and white-reference standardization. In this context, using another nominally equivalent calibrated spectroradiometer would mainly test instrument-to-instrument equivalence rather than the biological and spectral separability addressed here. The relevant translational step is instead transferred from this high-resolution proximal reference to alternative sensing architectures with different spectral response functions, band widths, illumination geometries and sampling footprints, including compact multispectral sensors, line-scan or area-scan hyperspectral imaging systems, and handheld VIS–NIR modules.

The class design was also intentionally operational. The nine labels combined taxonomic sources, organ types and colour/phenotype states because ornamental screening is typically performed on marketable leaves and bracts rather than on a single organ or species. Thus, the results of this study support sample-level ornamental identification but do not reveal the relative causal contribution of taxonomy, organ identity, colour patterning and tissue texture. Moreover, because one representative reflectance curve was retained per sampled leaf or bract, the benchmark evaluates sample-level classification rather than pixel-level mapping of within-organ heterogeneity. This is particularly relevant for variegated and transitional tissues, where future imaging or multi-point contact measurements could resolve sector-specific spectra.^3,58,62^

Finally, the present work should not be interpreted as a chemometric calibration for pigment, water, biochemical or anatomical traits. Those traits are discussed only as optical context from established leaf-reflectance theory and not as predicted response variables. The classification claim is instead supported by balanced plant-level sampling, an independent held-out test set and direct comparisons among spectral representations. Within this scope, the central result remains clear: full VIS–NIR–SWIR and edge-trimmed spectra retained the strongest performance, with macro-F1 = 82.56% and 81.99%, respectively, whereas the performance of the 16-band 547–562 nm window decreased to 60.77%. This confirms that the green window is informative but not a stand-alone replacement for broader spectral coverage.

## Supplementary Material



## Notes

Pearson correlations among class-mean spectra of the nine ornamental classes (Figure S1); supervised wavelength ranking using ANOVA effect size and ReliefF (Figure S2); spectral quality-control and edge-band trimming diagnostics (Figure S3); development-stage versus independent-test macro-F1 and balanced accuracy (Figure S4); baseline optimization trajectories and ROC and precision-recall curves (Figure S5); global permutation and SHAP wavelength importance for the reduced-band configuration (Figure S6); independent-test confusion matrices for reduced-band classifiers #01–#05 (Figure S7); independent-test confusion matrices for reduced-band classifiers #11–#15 (Figure S8); independent-test confusion matrices for reduced-band classifiers #16–#20 (Figure S9); independent-test confusion matrices for reduced-band classifiers #21–#25 (Figure S10); microaveraged one-versus-rest ROC and precision-recall curves for the main benchmark models (Figure S11); test macro-F1 across spectral representations and identification of the best-performing models (Figure S12); model-specific permutation-importance profiles for the first reduced-band classifier subset (Figure S13); model-specific permutation-importance profiles for the second reduced-band classifier subset (Figure S14); model-specific permutation-importance profiles for the third reduced-band classifier subset (Figure S15); model-specific mean absolute SHAP profiles for the first reduced-band classifier subset (Figure S16); model-specific mean absolute SHAP profiles for the second reduced-band classifier subset (Figure S17); model-specific mean absolute SHAP profiles for the third reduced-band classifier subset (Figure S18); taxonomic, organ, and phenotype descriptors of the nine ornamental classes (Table S1); selected wavelengths with ranking scores, SHAP importance, coefficient of variation, and edge-band status (Table S2); independent-test micro- and macroaveraged ROC-AUC and average-precision summaries for the principal reduced-band models (Table S3); bootstrap confidence intervals for independent-test accuracy, macro-F1, and balanced accuracy across spectral representations (Table S4); paired comparisons among spectral representations using exact McNemar tests and bootstrap macro-F1 differences (Table S5) (PDF)
