# Supplementary material for: VIS–NIR–SWIR Proximal Spectroradiometry Coupled with Machine Learning and Deep Learning for Ornamental Plant Identification and Classification
Source: ACS Omega. 2026 Sep 14;11(38):57197–212. doi: 10.1021/acsomega.6c05120 (PMC13628991; doi:10.1021/acsomega.6c05120)
Supplement: Supplementary file 1 [file ao6c05120_si_001.pdf]

Supplementary files

VIS–NIR–SWIR Proximal Spectroradiometry Coupled with Machine Learning and Deep Learning for Ornamental Plant Identification and Classification

Renan Falcioni[1,2*], José Alexandre M. Demattê[2] and Marcos Rafael Nanni[1]

[1] Graduate Program in Agronomy, State University of Maringá, Av. Colombo, 5790, Maringá 87020−900, Paraná, Brazil

[2] Department of Soil Science, Luiz de Queiroz College of Agriculture, University of São Paulo, Av. Pádua Dias, 11, Piracicaba 13418−260, São Paulo, Brazil

[*]Correspondence:
Renan Falcioni; renanfalcioni@gmail.com or rfalcioni2@uem.br
Phone: +55 44 3011−1359

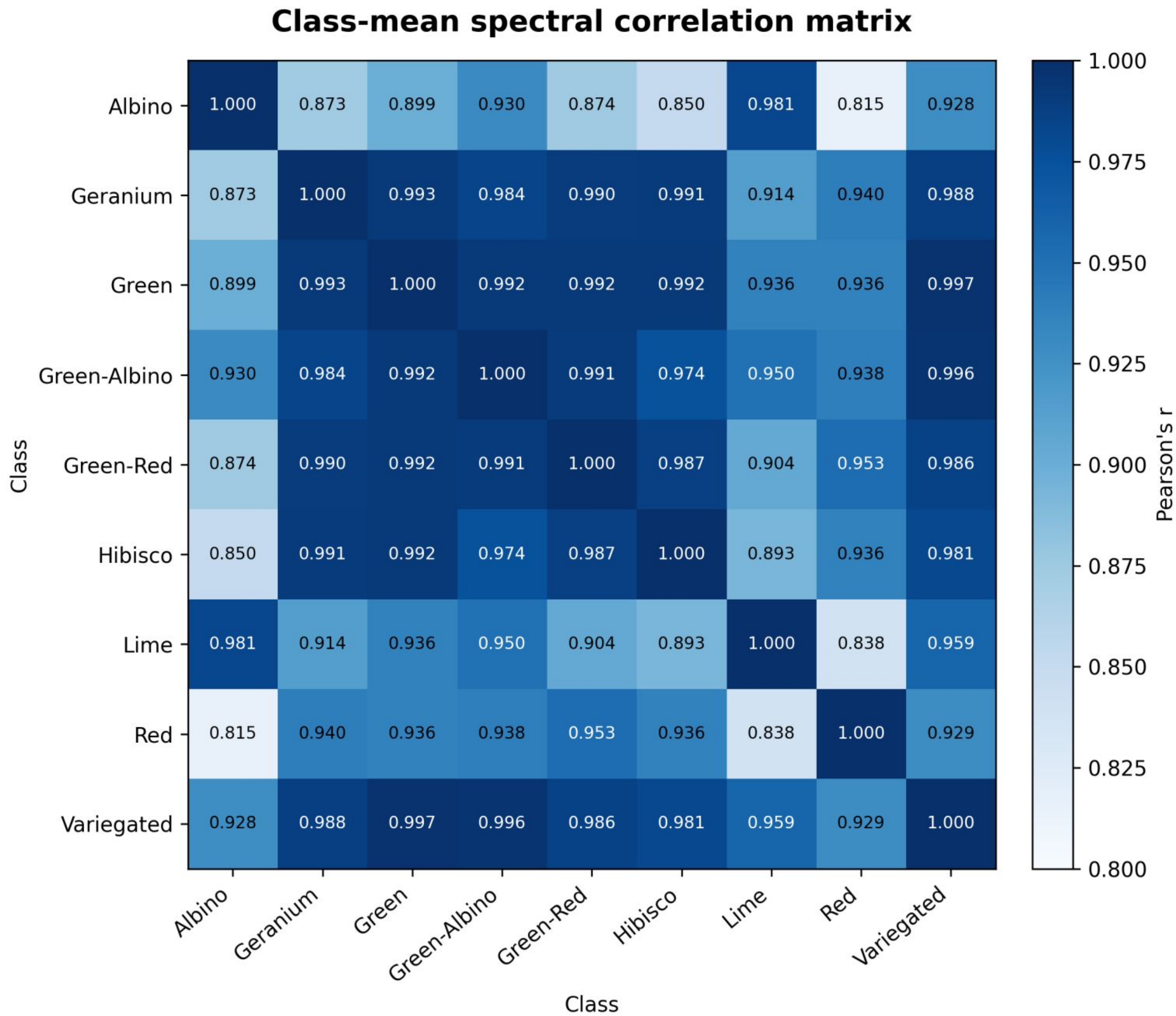

Figure S1. Correlation matrix of the class-mean spectra across the nine ornamental classes. The cell values represent pairwise Pearson correlation coefficients among pre-processed class means and summarise global spectral similarity across the benchmark, highlighting both the crowded green-centred neighbourhoods and the more isolated high-contrast classes.

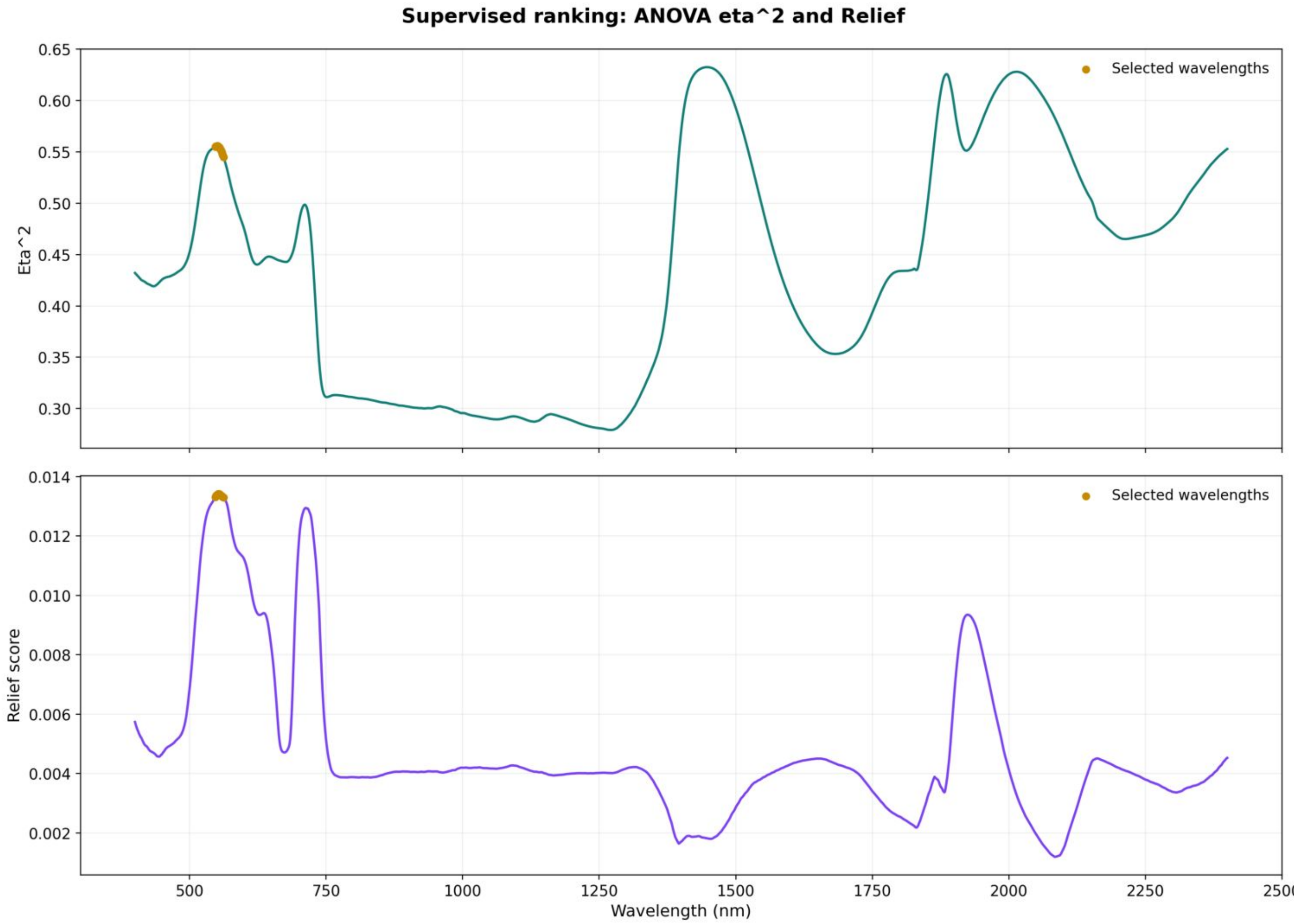

Figure S2. Supervised wavelength ranking based on ANOVA eta^2 and ReliefF. The figure compares the global between-class effect size with the local multiclass feature relevance and highlights the wavelengths retained in the reduced-band ornamental benchmark.

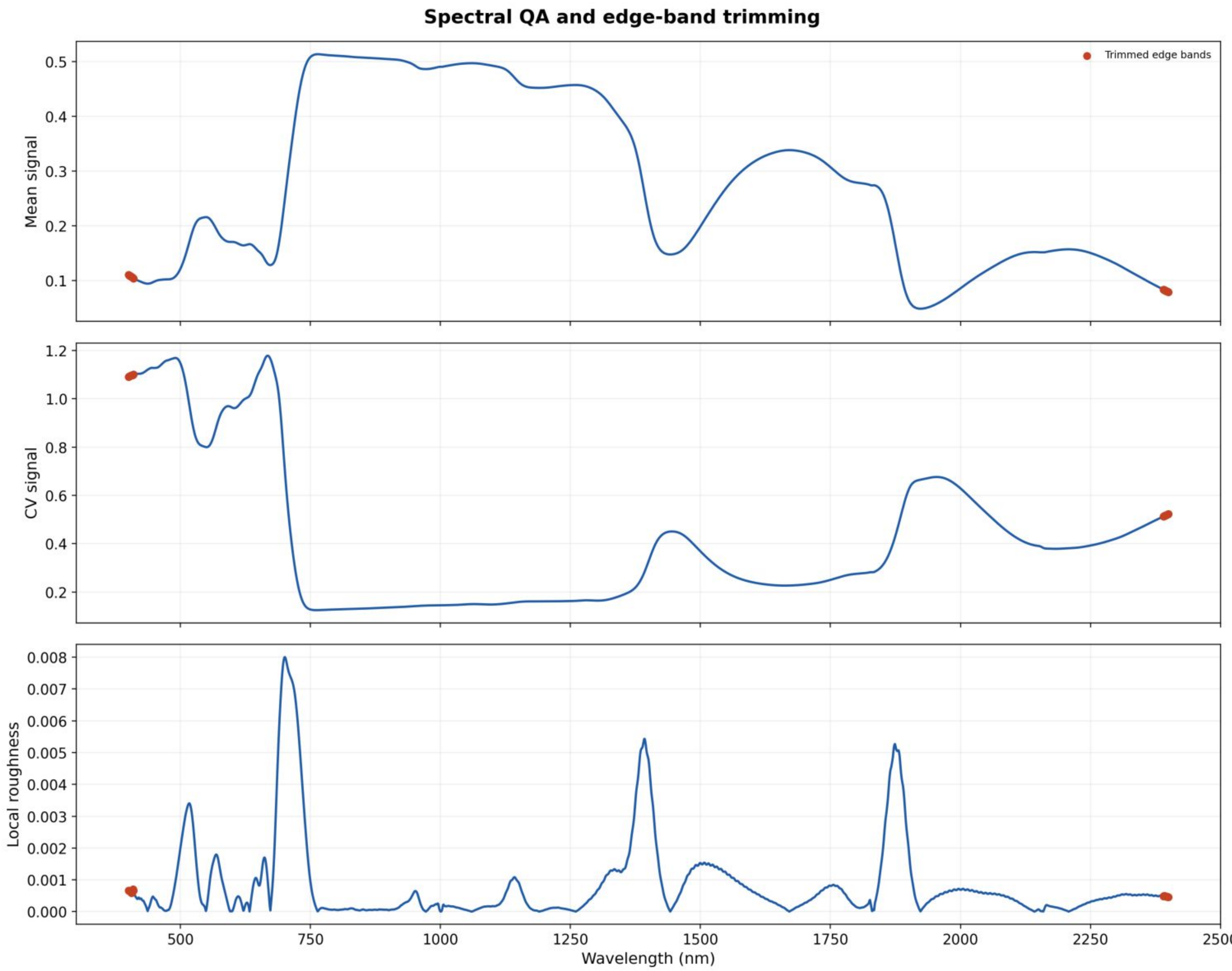

Figure S3. Spectral quality-control summary and edge-band trimming rationale. Panels show the mean signal, coefficient of variation, local roughness and placement of the selected wavelengths over the trimmed mean spectrum, explaining why unstable spectral-edge regions were removed before reduced-band analysis.

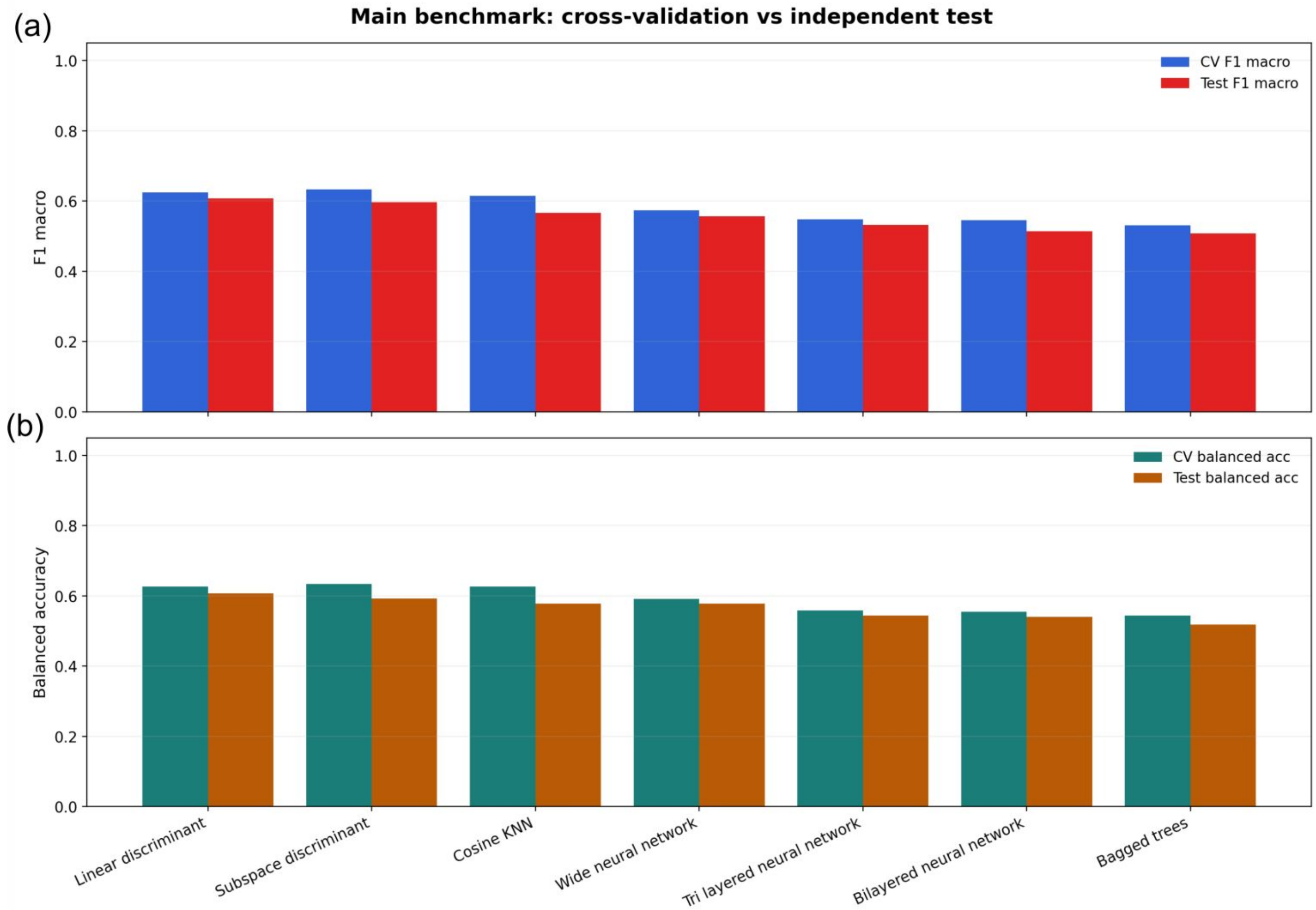

Figure S4. Agreement between development-stage and independent-test performance in the main classical benchmark. Panel (a) shows macro-F1 and panel (b) balanced accuracy for representative compact models, allowing direct visualisation of the generalisation gap between tuning and held-out evaluation.

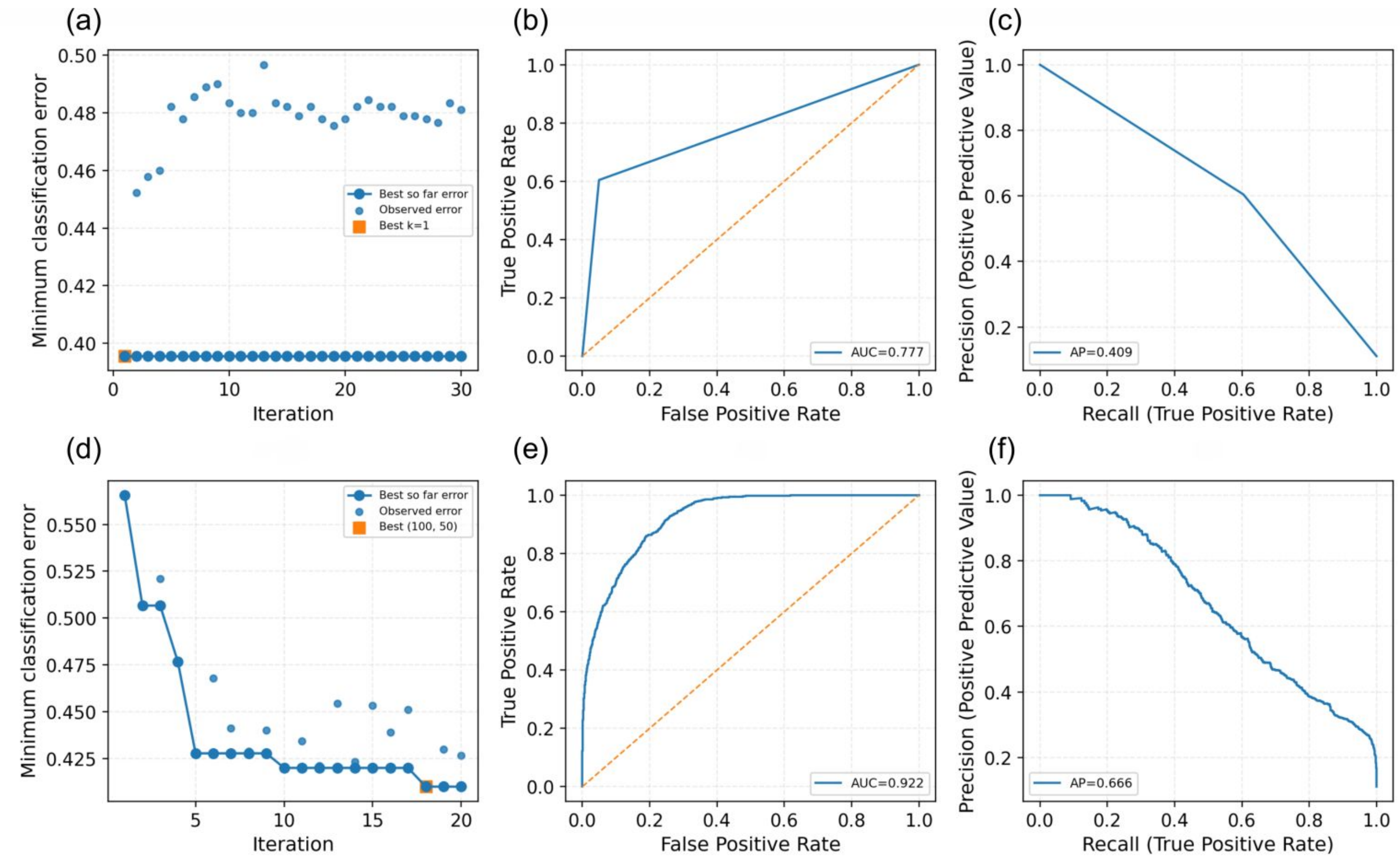

Figure S5. Internal optimisation diagnostics and ranking-quality summaries for representative baseline workflows. (a) Development-set tuning trajectory for the first workflow; (b) corresponding receiver operating characteristic (ROC) curve (AUC = 0.777); (c) corresponding precision-recall curve (AP = 0.409); (d) development-set tuning trajectory for the second workflow; (e) corresponding ROC curve (AUC = 0.922); and (f) corresponding precision-recall curve (AP = 0.666). These diagnostics were used to compare probabilistic ranking quality beyond hard class assignment.

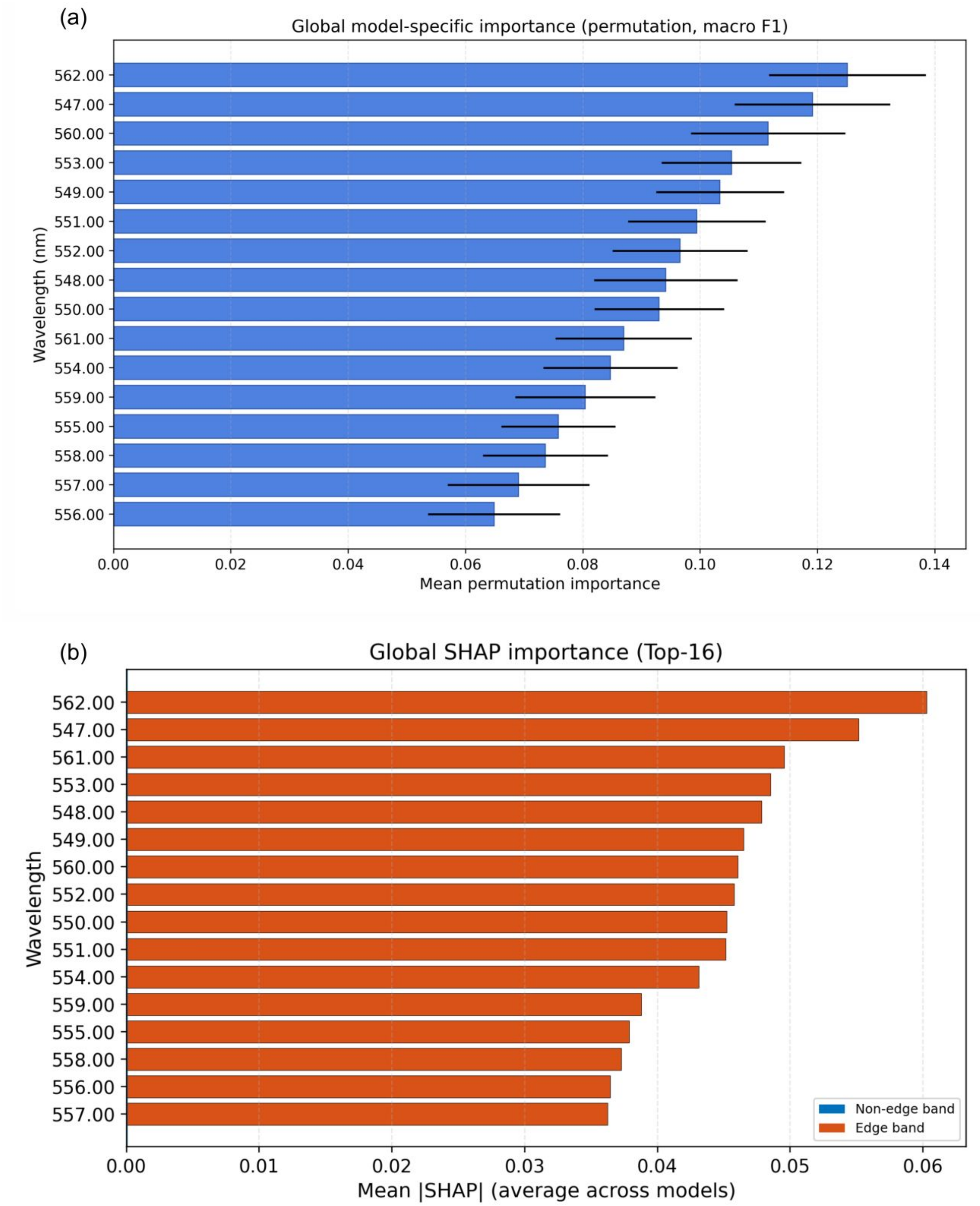

Figure S6. Global permutation and SHAP attribution for the reduced-band ornamental configuration. Panel (a) shows the mean macro-F1-based permutation importance across compact models, and panel (b) shows the mean absolute SHAP importance across models. Together, the panels identify the most influential wavelengths within the 547–562 nm reduced set.

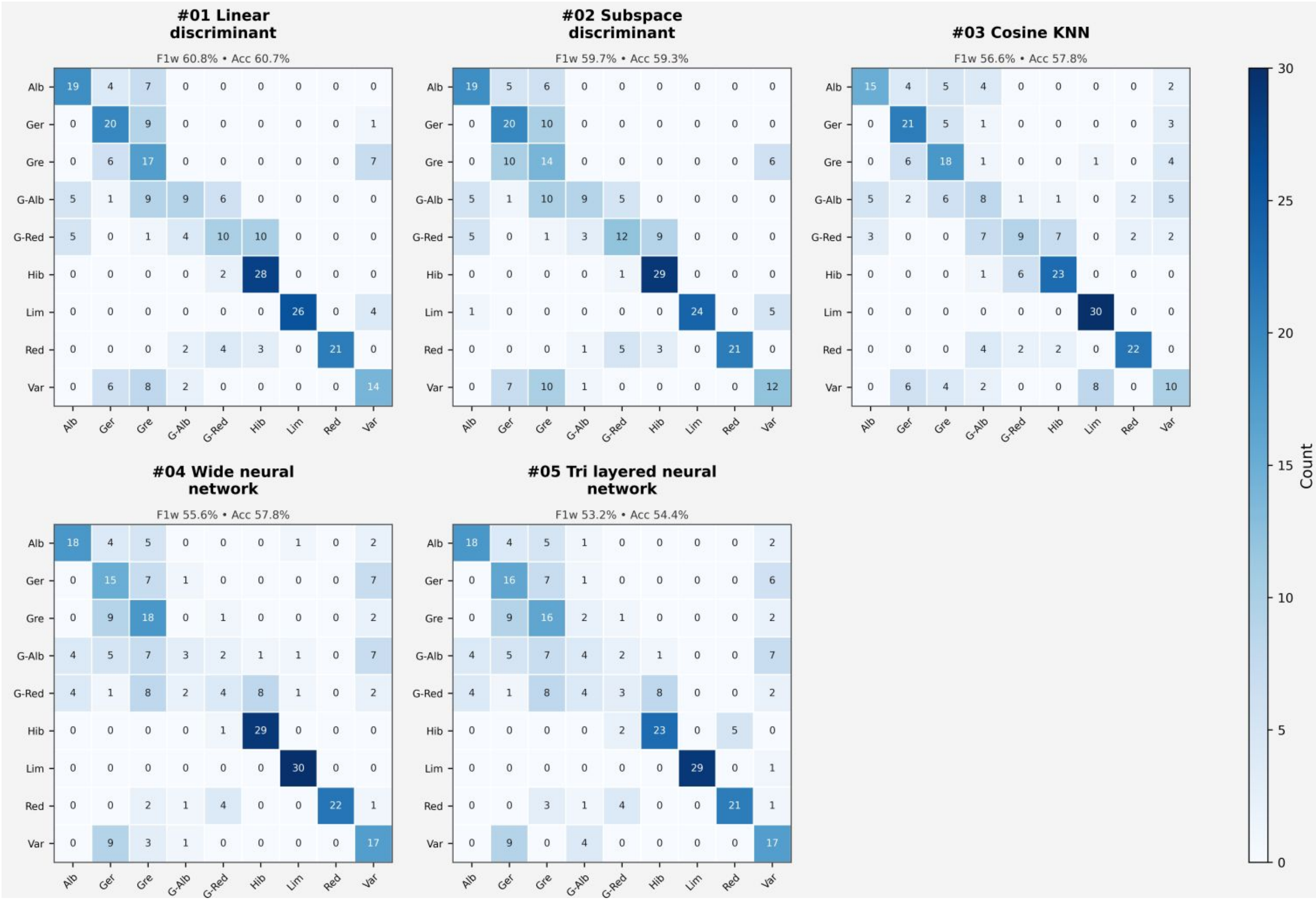

Figure S7. Independent-test confusion matrices for the first subset of reduced-band classifiers (#01-#05), including the highest-ranked compact models. Rows correspond to true classes and columns to predicted classes; cell values are counts from the 270 held-out plant-level test samples.

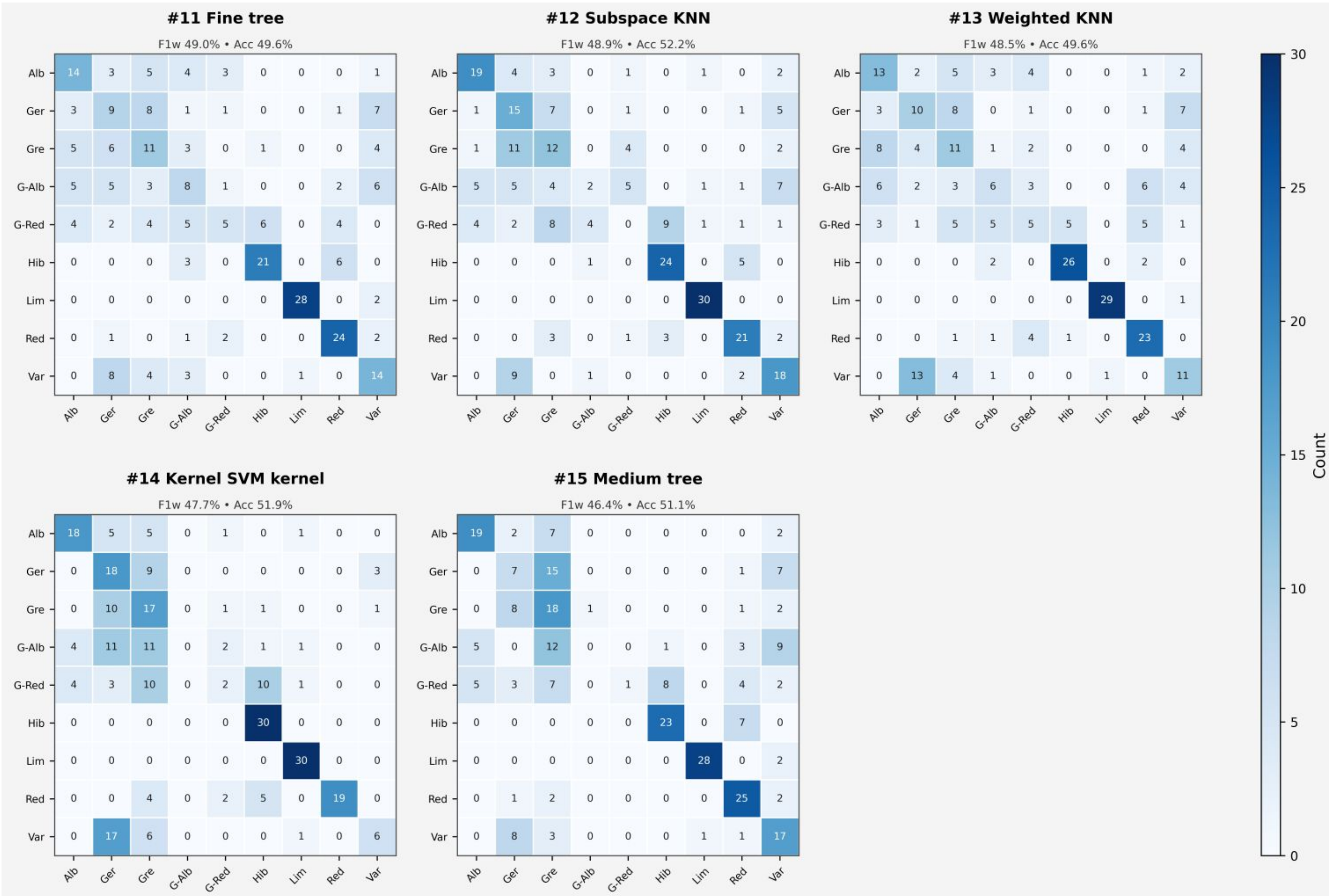

Figure S8. Independent-test confusion matrices for an intermediate subset of reduced-band classifiers (#11-#15). The panels illustrate how performance degradation in the compact 547–562 nm representation is expressed as structured confusion among green-centred ornamental phenotypes.

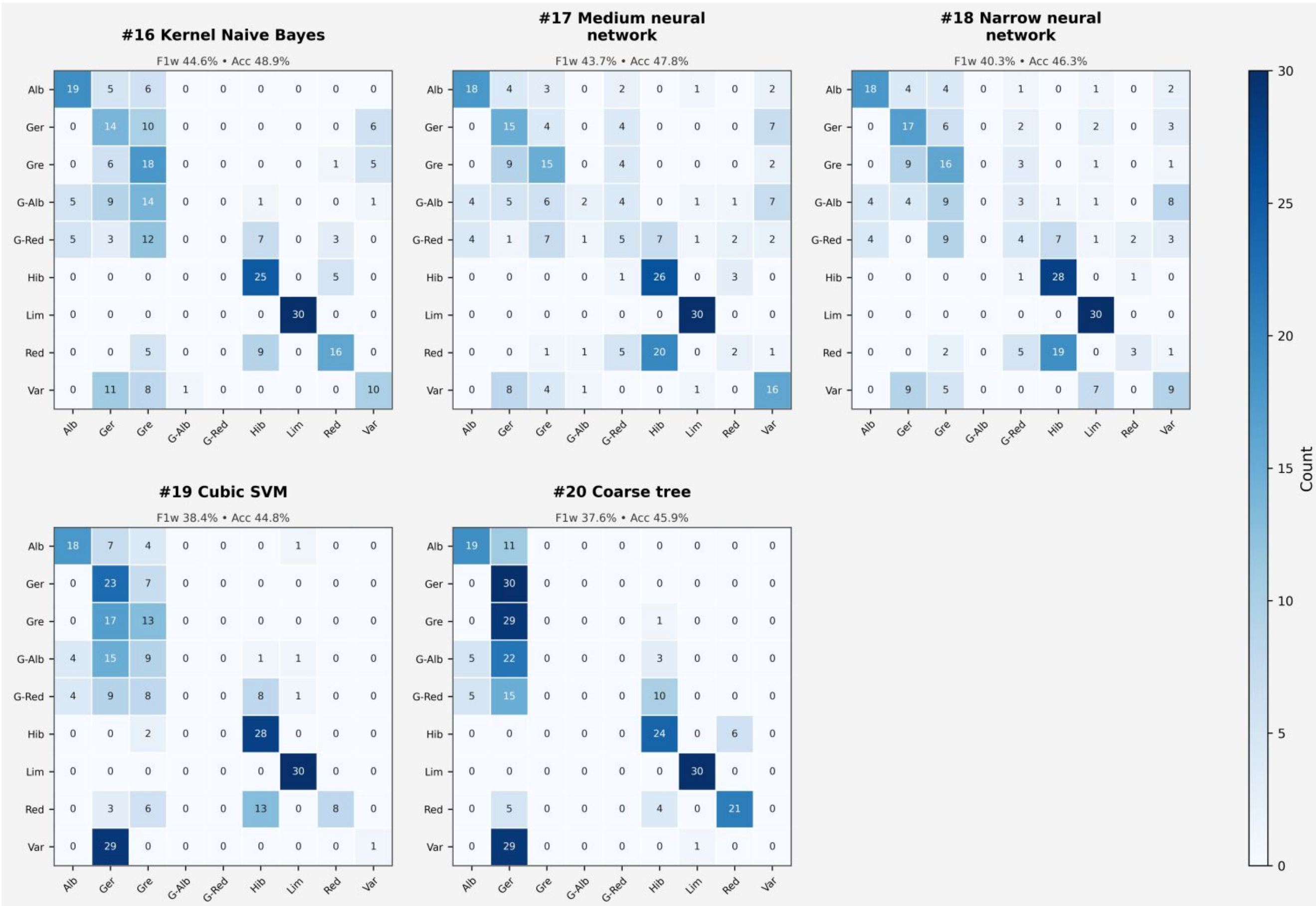

Figure S9. Independent-test confusion matrices for a lower-performing subset of reduced-band classifiers (#16-#20). The panels show increasing off-diagonal concentrations as the model performance decreases under the compact 16-band representation.

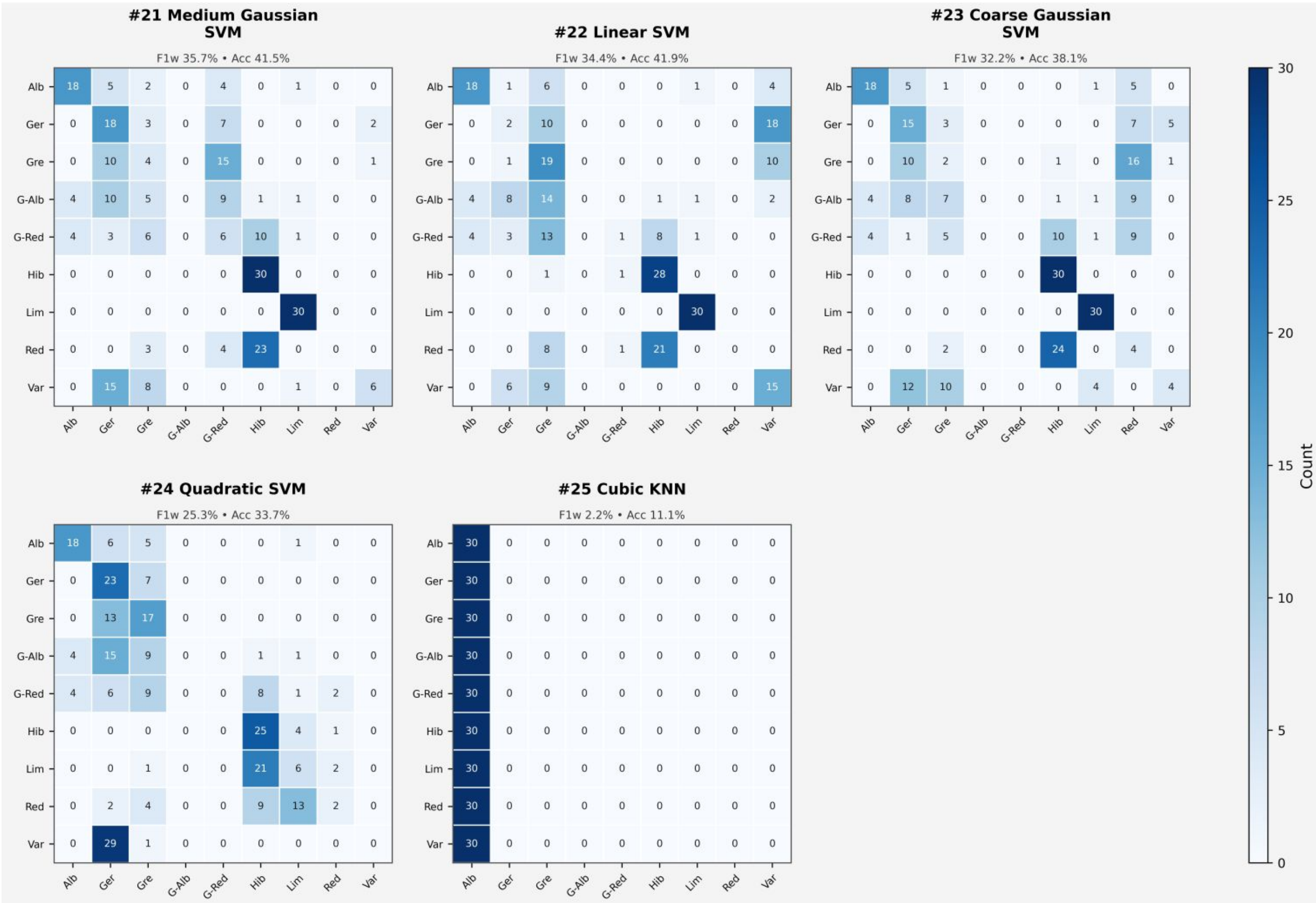

Figure S10. Independent-test confusion matrices for the lowest-performing subset of reduced-band classifiers (#21-#25). The panels emphasise that severe model underperformance was not random but often involved collapse toward a few visually and spectrally dominant classes.

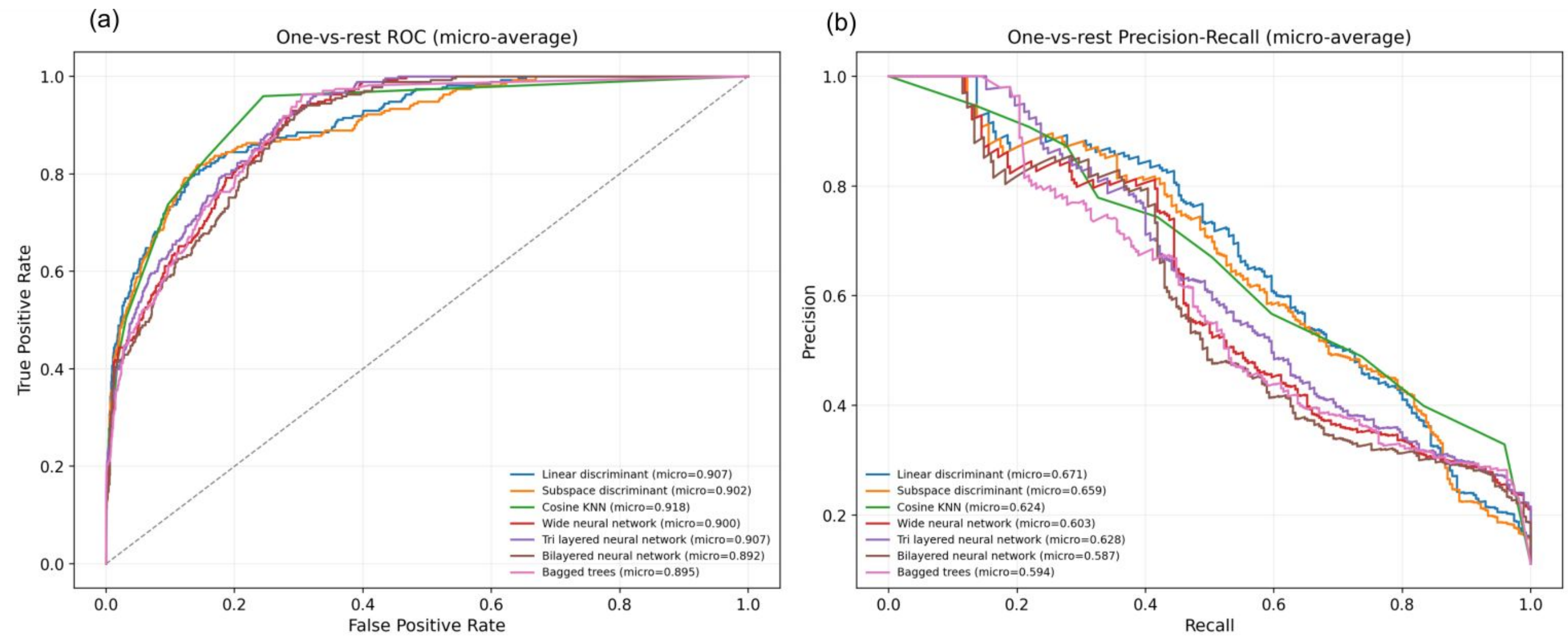

Figure S11. Microaveraged one-versus-rest ROC and precision–recall curves for the main ornamental benchmark models. The curves summarise the ranking quality across all classes and complement the hard-classification metrics reported in the main text.

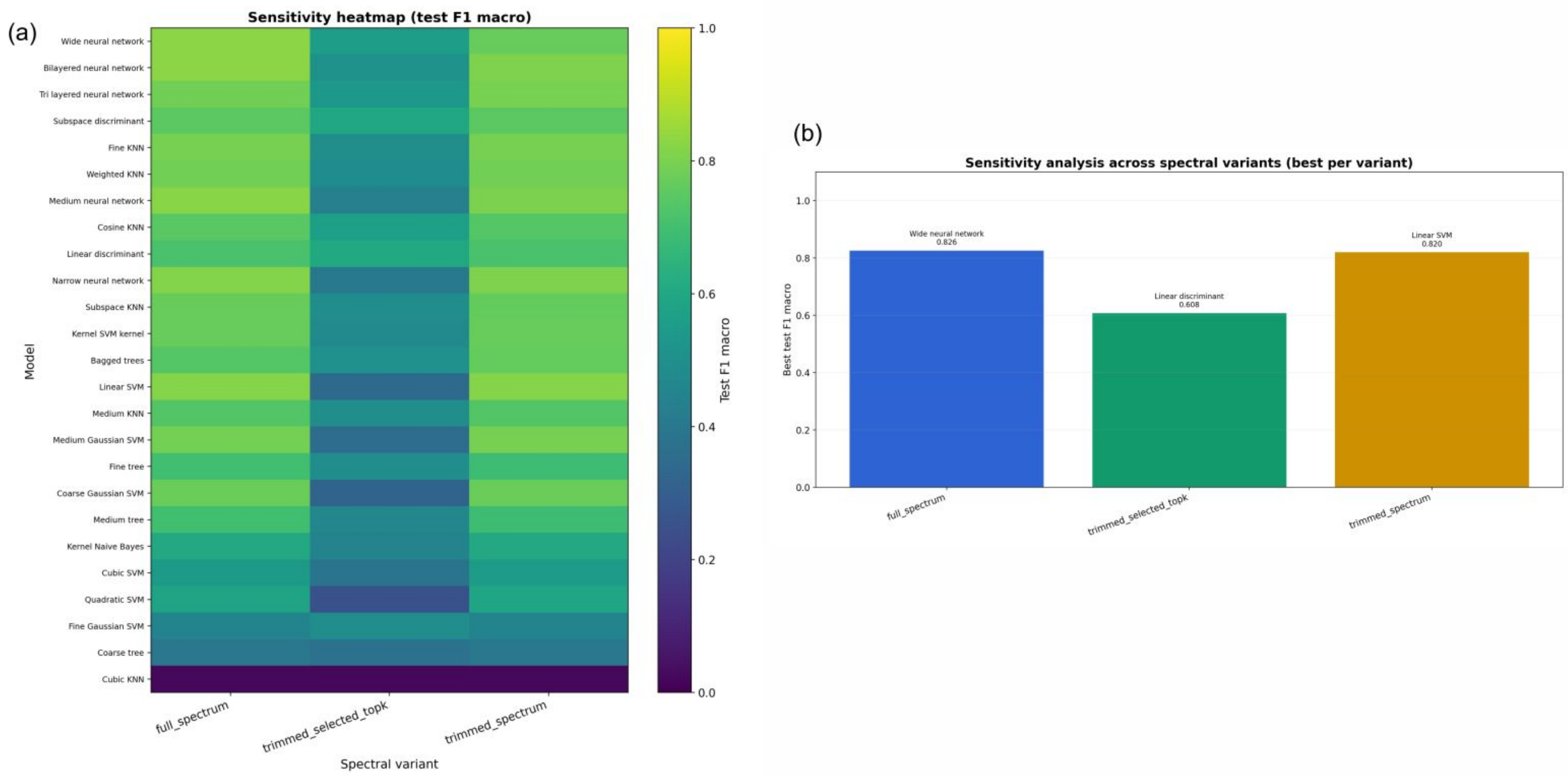

Figure S12. Heatmap summary of model sensitivity across ornamental spectral variants and comparison of the best score obtained within each variant. Panel (a) shows the macro-F1 test results by model and spectral representation, whereas panel (b) identifies the top-performing model for each variant.

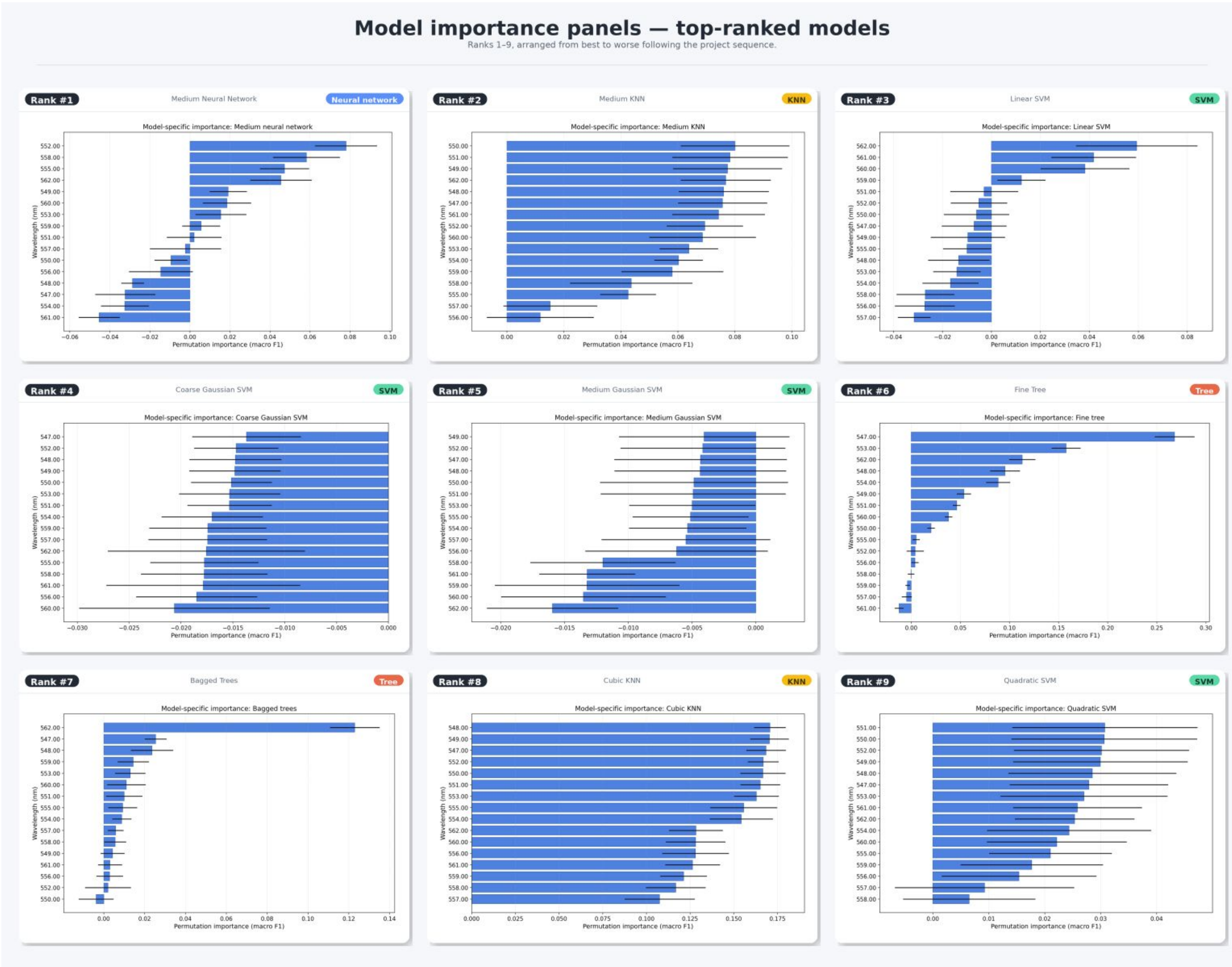

Figure S13. Model-specific permutation-importance profiles for the first subset of reduced-band classifiers. The bars show the macro-F1-based permutation importance for each selected wavelength and allow comparison of the local attribution structure against the global ranking.

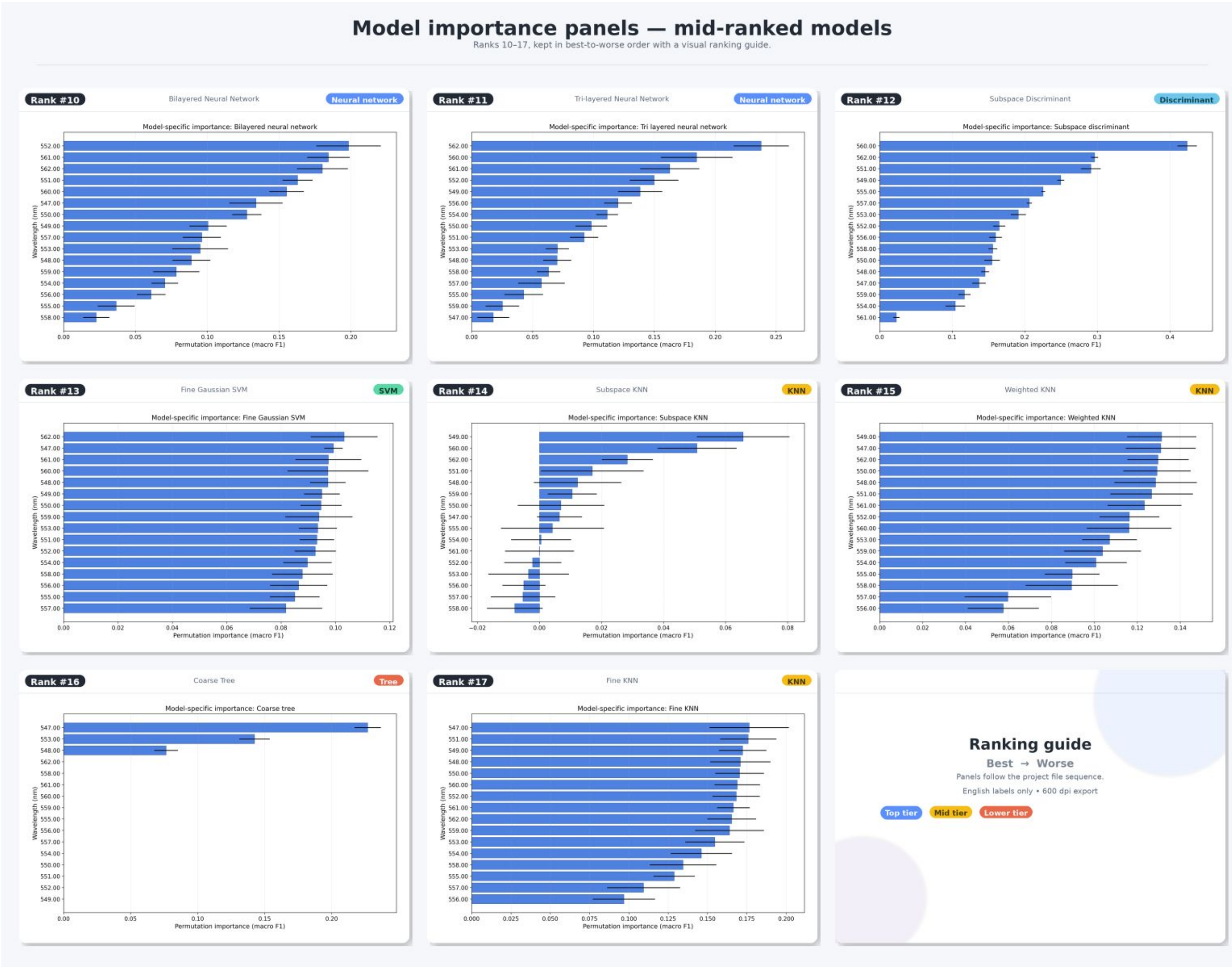

Figure S14. Model-specific permutation-importance profiles for the second subset of reduced-band classifiers. The bars show the importance of macro-F1-based permutations for each selected wavelength and highlight models with high concordance with the global wavelength ranking.

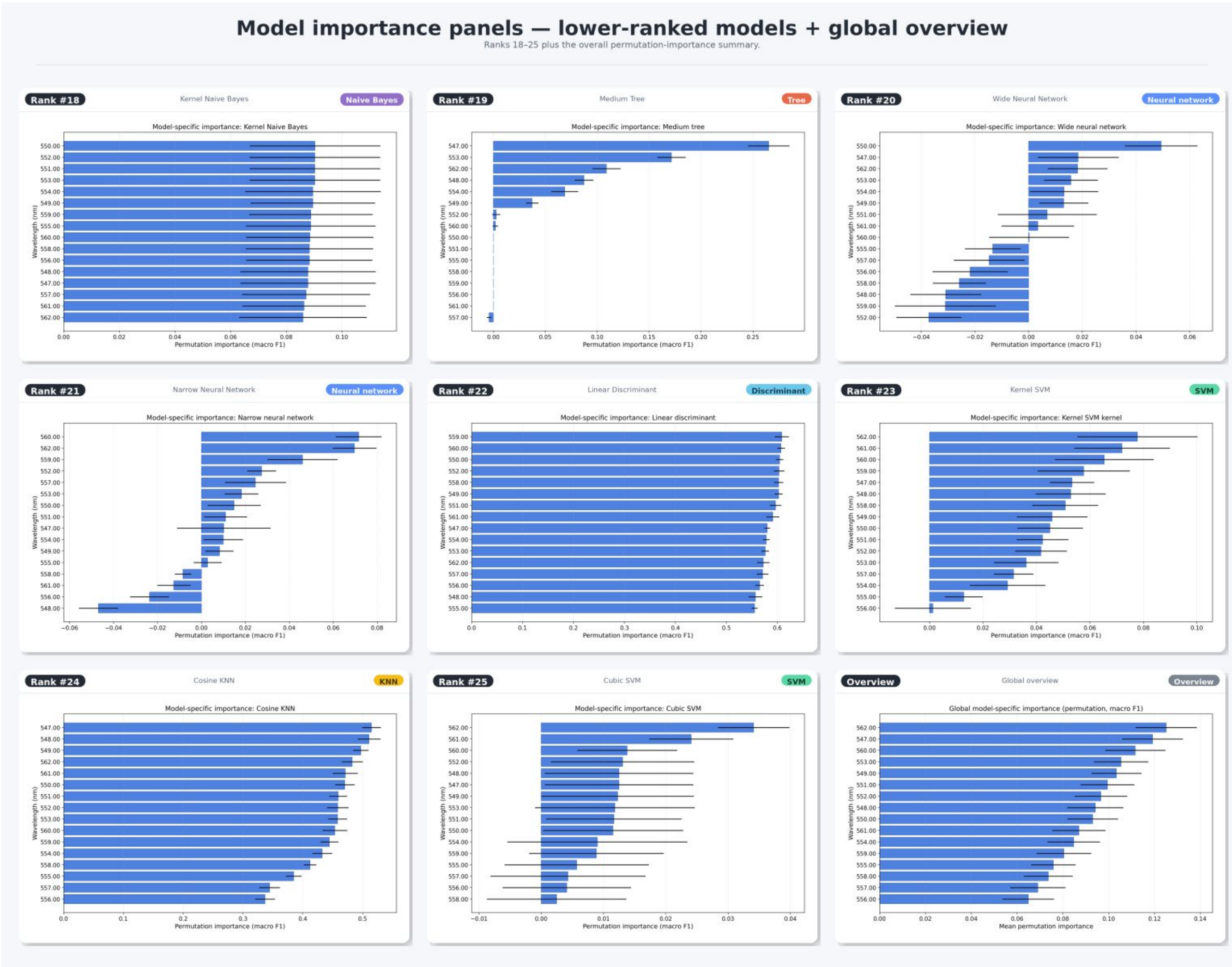

Figure S15. Model-specific permutation-importance profiles for the third subset of reduced-band classifiers. The bars show the importance of macro-F1-based permutations for each selected wavelength and highlight models with more intermediate or hybrid attribution structures.

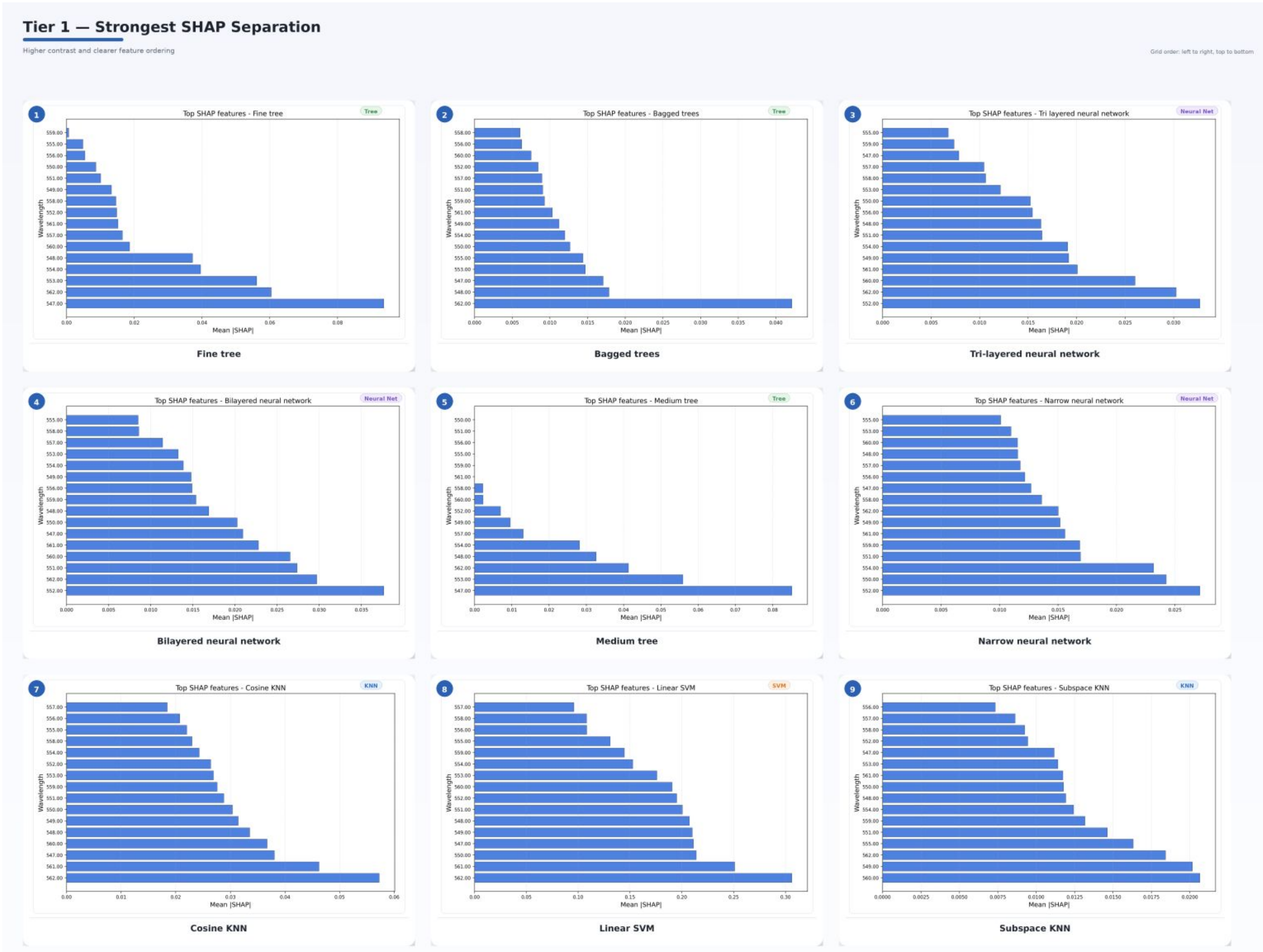

Figure S16. Model-specific SHAP profiles for the first subset of reduced-band classifiers. The bars represent the mean absolute SHAP values across the selected wavelengths and show how local model explanations align with the global importance pattern.

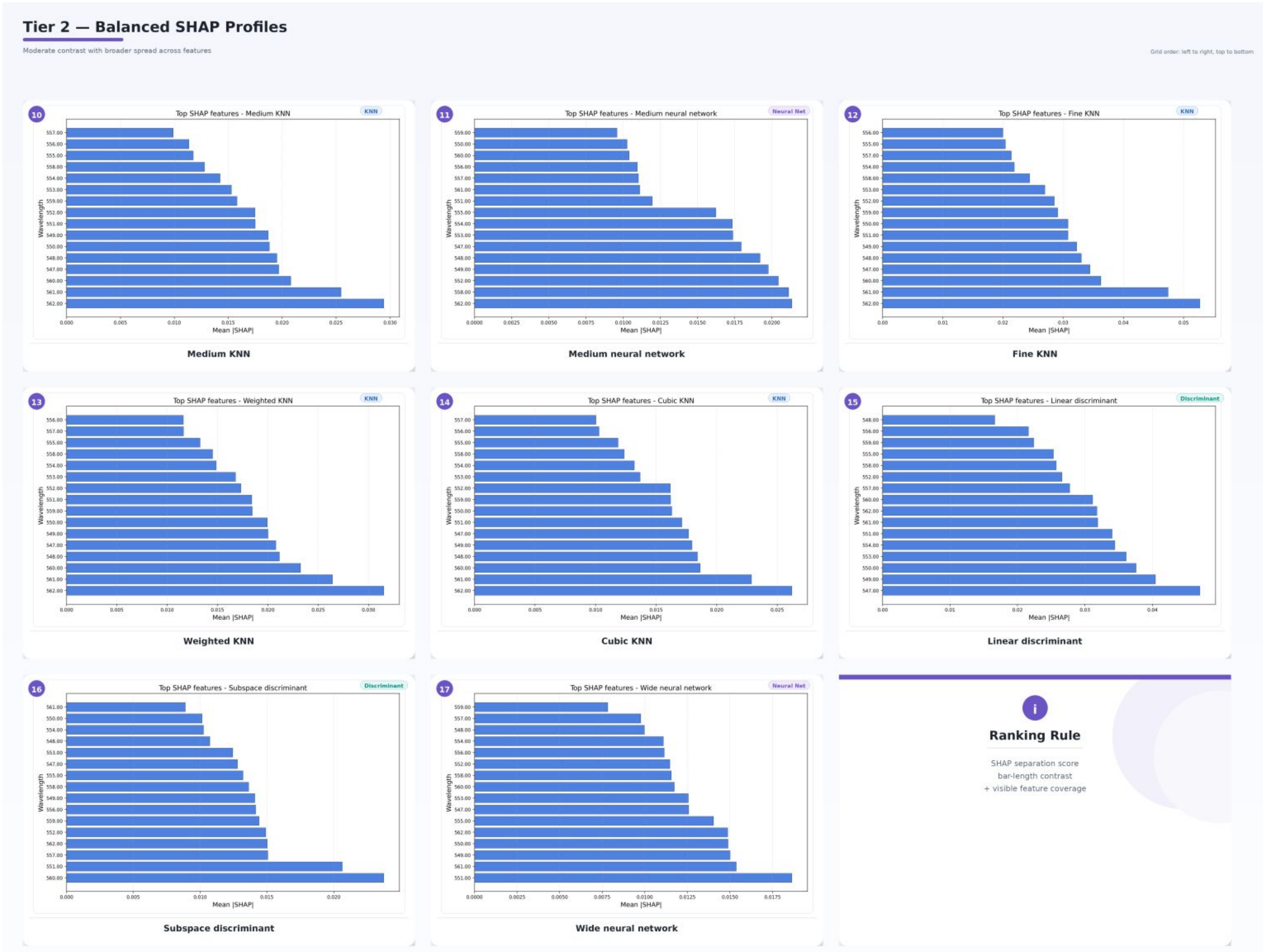

Figure S17. Model-specific SHAP profiles for the second subset of reduced-band classifiers. The bars represent the mean absolute SHAP values across the selected wavelengths and show models with high, although not identical, agreement with the global importance pattern.

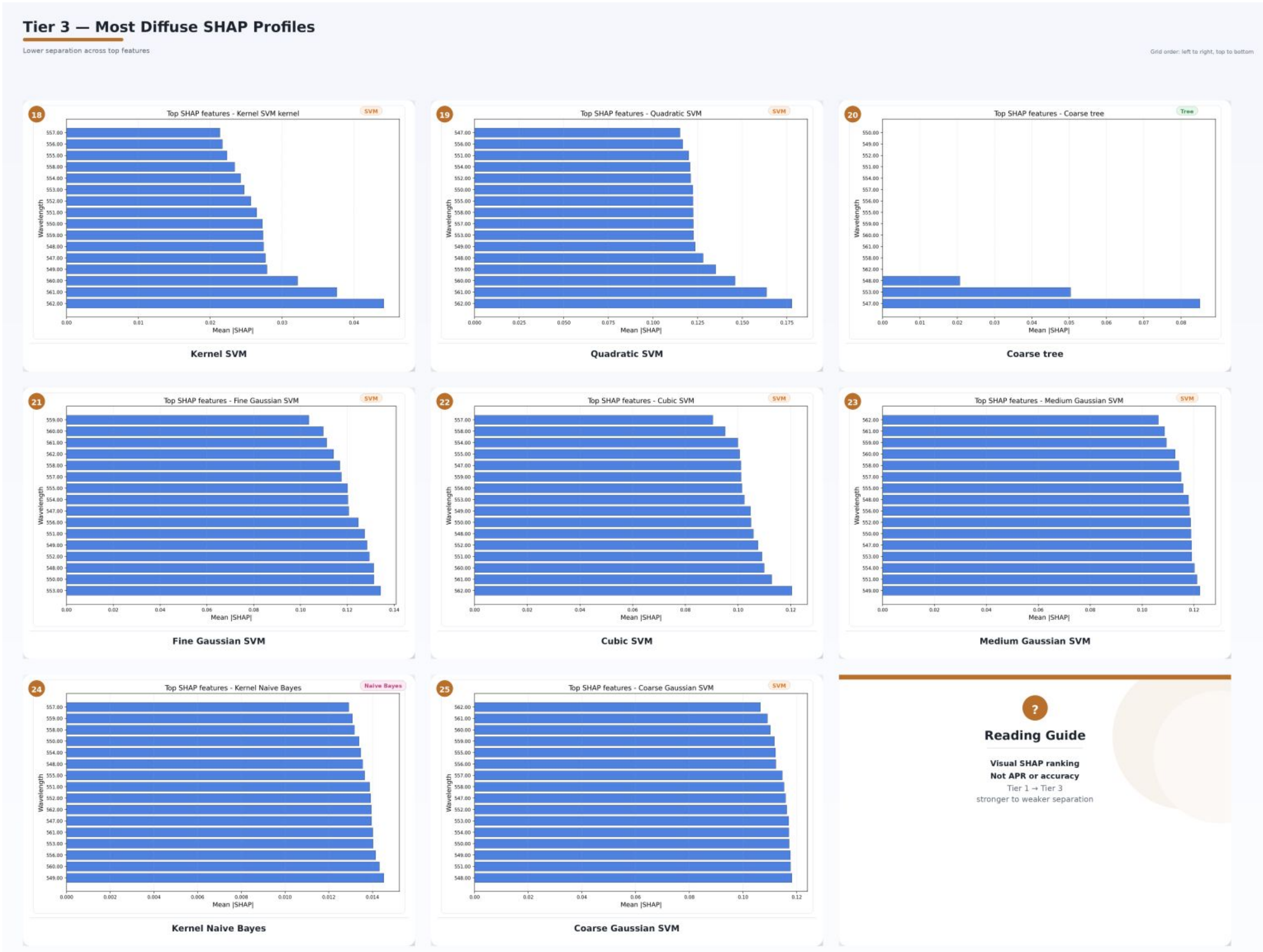

Figure S18. Model-specific SHAP profiles for the third subset of reduced-band classifiers. The bars represent the mean absolute SHAP values across the selected wavelengths and show models with intermediate selectivity and partially divergent attribution structures.

Table S1. Complete descriptor matrix of the nine ornamental classes analysed in this study, including taxonomic source, common name, organ, class label and phenotype definition.

Species	Common name	Class label	Descriptor	Organ
Euphorbia pulcherrima	Poinsettia	Albino	Near-white/cream ornamental bracts with very low visible chlorophyll.	Bract
Pelargonium zonale	Geranium	Geranium	Rounded to slightly lobed medium-green leaves with mild pubescence.	Leaf
Epipremnum aureum	Pothos	Green	Uniform dark-green, chlorophyll-rich Jade-type foliage.	Leaf
Epipremnum aureum	Pothos	Green-Albino	Sectorial foliage with green tissue and broad cream to near-white patches.	Leaf
Euphorbia pulcherrima	Poinsettia	Green–Red	Transitional ornamental tissue retaining green areas while red pigmentation develops.	Bract
Hibiscus rosa-sinensis	Hibiscus	Hibisco	Broad glossy green leaves with a smooth surface.	Leaf
Epipremnum aureum	Pothos	Lime	Uniform lime-green Neon-type foliage with reduced apparent chlorophyll.	Leaf
Euphorbia pulcherrima	Poinsettia	Red	Fully pigmented red ornamental bracts with minimal residual green.	Bract
Epipremnum aureum	Pothos	Variegated	Green–yellow-cream marbled Golden-type foliage.	Leaf

Table S2. Wavelengths selected by supervised ranking in the trimmed ornamental benchmark, with ReliefF score, ANOVA eta^2, mean absolute SHAP value across models, coefficient of variation and edge-band status.

Wavelength (nm)	Region	Relief score	ANOVA eta^2	Mean |SHAP|	CV (%)	Edge band
547	VIS green–yellow (500–700)	0.0133	0.5552	0.0551	80.13	No
548	VIS green–yellow (500–700)	0.0133	0.5555	0.0479	80.08	No
549	VIS green–yellow (500–700)	0.0134	0.5557	0.0465	80.03	No
550	VIS green–yellow (500–700)	0.0134	0.5558	0.0452	80.01	No
551	VIS green–yellow (500–700)	0.0134	0.5557	0.0451	80.01	No
552	VIS green–yellow (500–700)	0.0134	0.5555	0.0458	80.05	No
553	VIS green–yellow (500–700)	0.0134	0.5552	0.0485	80.14	No
554	VIS green–yellow (500–700)	0.0134	0.5546	0.0431	80.28	No
555	VIS green–yellow (500–700)	0.0134	0.5539	0.0379	80.46	No
556	VIS green–yellow (500–700)	0.0134	0.5530	0.0364	80.71	No
557	VIS green–yellow (500–700)	0.0134	0.5520	0.0362	81.01	No
558	VIS green–yellow (500–700)	0.0134	0.5508	0.0373	81.37	No
559	VIS green–yellow (500–700)	0.0133	0.5495	0.0388	81.78	No
560	VIS green–yellow (500–700)	0.0133	0.5481	0.0460	82.25	No
561	VIS green–yellow (500–700)	0.0133	0.5467	0.0496	82.77	No
562	VIS green–yellow (500–700)	0.0133	0.5451	0.0602	83.34	No

Table S3. Micro- and macroaveraged one-versus-rest ROC-AUC and average-precision (AP) summaries for the principal reduced-band ornamental benchmark models evaluated on the independent test set.

Model	ROC-AUC (micro)	ROC-AUC (macro)	AP (micro)	AP (macro)
Linear discriminant	0.907	0.903	0.671	0.654
Subspace discriminant	0.902	0.902	0.659	0.654
Cosine KNN	0.918	0.908	0.624	0.596
Wide neural network	0.900	0.875	0.603	0.558
Tri layered neural network	0.907	0.877	0.628	0.559
Bilayered neural network	0.892	0.863	0.587	0.557
Bagged trees	0.895	0.871	0.594	0.545

Table S4. Bootstrap uncertainty for the representative representation-level validation performed from the Plantinhas.xlsx workbook under the same fixed 35:35:30 plant-level split. The values are independent-test percentages with 95% confidence intervals from 10,000 non-parametric bootstrap resamples of the 270 held-out samples.

Representation	Representative model	Accuracy, % (95% CI)	Macro-F1, % (95% CI)	Balanced accuracy, % (95% CI)	Correct/test samples
Full spectrum	Linear SVM	82.59 (77.78-87.04)	82.63 (78.07-86.58)	82.59 (78.43-86.59)	223/270
Trimmed spectrum	Linear SVM	81.85 (77.04-86.30)	81.90 (77.35-85.82)	81.85 (77.58-85.86)	221/270
16-band green window	LDA	60.74 (54.81-66.30)	60.77 (55.00-65.85)	60.74 (55.36-66.12)	164/270

Table S5. Paired statistical comparisons among the representative spectral representations are shown in Table S4. Discordant counts are reported as A-correct/B-incorrect versus A-incorrect/B-correct. The McNemar P values are exact two-sided tests; macro-F1 differences are bootstrap mean differences in percentage points with 95% confidence intervals.

**Paired comparison**	**Discordant counts**	**Exact McNemar P**	**Macro-F1 difference, percentage points (95% CI)**
Full spectrum vs Trimmed spectrum	2/0	0.500	0.73 (0.00-1.86)
Full spectrum vs 16-band green window	66/7	<0.001	22.01 (16.73-27.21)
Trimmed spectrum vs 16-band green window	64/7	<0.001	21.25 (15.99-26.33)
